# Supplementary material for: Stage-resolved Hi-C analyses reveal meiotic chromosome organizational features influencing homolog alignment
Source: Nat Commun. 2021 Oct 8;12:5827. doi: 10.1038/s41467-021-26033-0 (PMC8501046; doi:10.1038/s41467-021-26033-0)
Supplement: Supplementary file 1 — Supplementary Information [file 41467_2021_26033_MOESM1_ESM.pdf]

Supplementary Information

Stage-resolved Hi-C Analyses Reveal Meiotic Chromosome Organizational Features Influencing Homolog Alignment

Wu Zuo, Guangming Chen, Zhimei Gao *et al*.

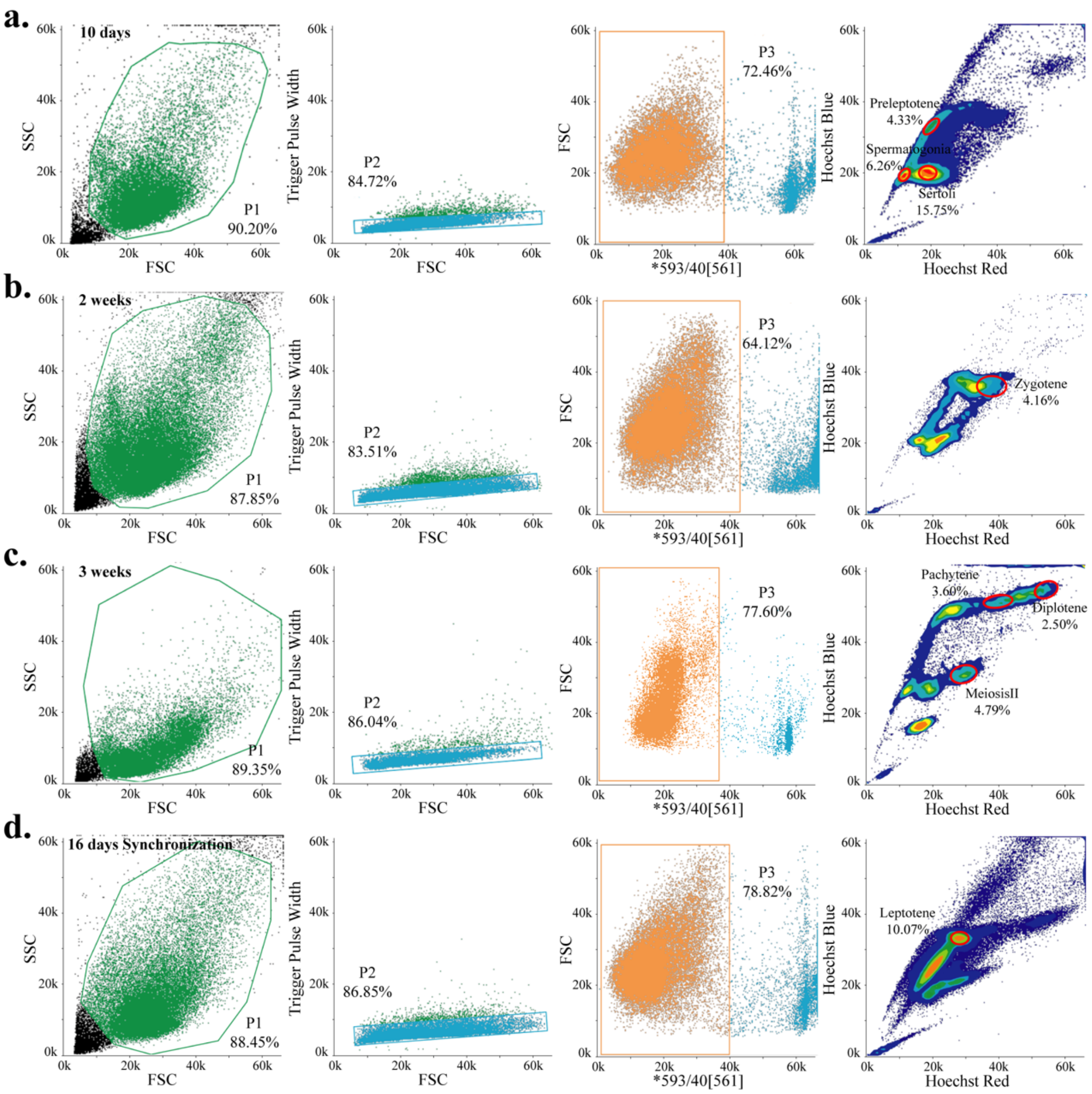

Supplementary Figure 1. Gating strategy to isolation of somatic cells and spermatocytes

a-d, Representative FACS sequential gating strategy profiles show the isolation of different cell types in mice of different ages that correspond to the FACS data panels in Figure 1b-e. Debris was excluded based on the FSC and SSC parameters and intact cells were defied as Population 1 (P1), afterward Trigger Pulse Width parameter was applied to the P1 to exclude cytoadherence cells and the remaining single cells were defied as Population 2 (P2). The PI-negative population was defined as living cells and derived from P2, defined as Population 3 (P3). Finally, Hoechst profiles of P3 show the separation of different cell types by fluorescence intensity: 10 days old mice for isolating Sertoli, spermatogonia, and preleptotene cells (**a**), 2-week old mice for zygotene cells (**b**), 3 weeks old mice for pachytene, diplotene, and Meiosis II cells (**c**),

16 days synchronized mice for isolation of leptotene cells (**d**). Red circles in each profile indicate gating windows used for cell isolation. Relative proportions for each gate are indicated in the figure.

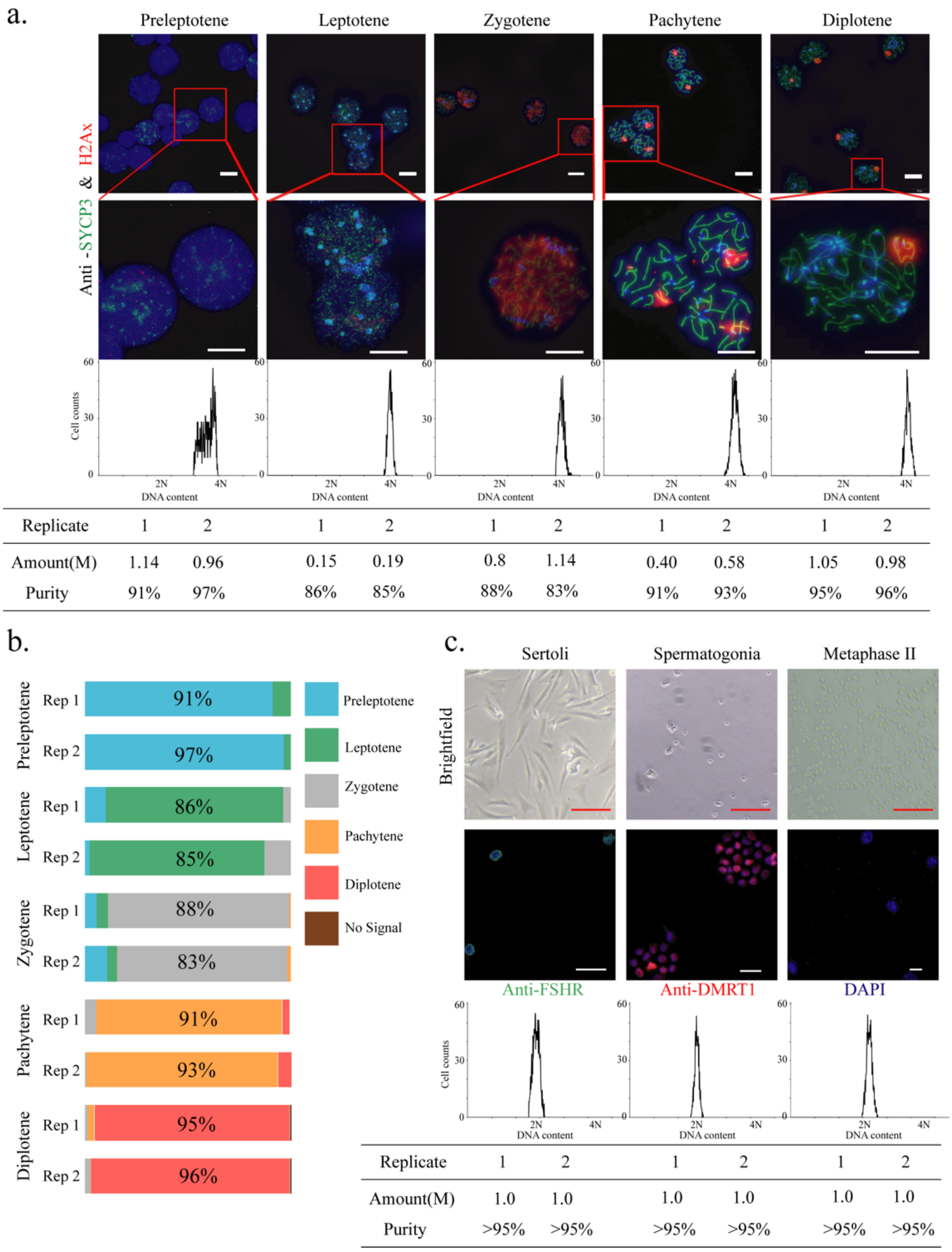

Supplementary Figure 2. Isolation of somatic cells and spermatocytes of precise stages for Hi-C analyses

a, Representative immunofluorescence (IF) images of isolated preleptotene, leptotene, zygotene, pachytene, and diplotene stage spermatocytes stained for meiosis markers SYCP3 (green) and H2Ax (red). DAPI-stained DNA is shown in blue. Scale bars of the upper row and lower row are 20 μm and 10 μm, respectively. For purity assessment, cells exhibiting weak, diffused or punctate SYCP3 but no obvious stretches of SYCP3 signals were categorized as preleptotene. Cells showing short to long SYCP3 stretches

that do not span over the entire length of the chromosome were categorized as leptotene. Cells exhibiting long SYCP3 fibers as well as strong γH2AX signals, but not exhibiting the XY sex body that is intensely labeled by γH2AX were categorized as zygotene. Cells exhibiting "stubby" SYCP3 signals that indicate the fully synapsed tetrad chromosomes, as well as the appearance of γH2AX-labelled sex body, were categorized as pachytene. Finally, cells exhibiting partially separated chromosome axes were categorized as diplotene.

b, Bar plot indicates the purity for each batch of purified spermatocytes. A small fraction of cells exhibited no DAPI staining and were categorized as "No Signal". The relevant information can be found in Supplementary Table 1.

c, Representative brightfield and IF images of isolated Sertoli, spermatogonia, and Meiosis II cells. Sertoli and Spermatogonia cells were stained with antibodies recognizing FSHR (green) and DMRT1 (red) proteins to confirm the cell types. DAPI-stained DNA is shown in blue. Scale bar for brightfield images, 100 μm. Scale bar for IF images, 50 μm.

Representative DNA ploidy profile along with the quantity and purity of each isolated cell population is indicated below the images in a and c.

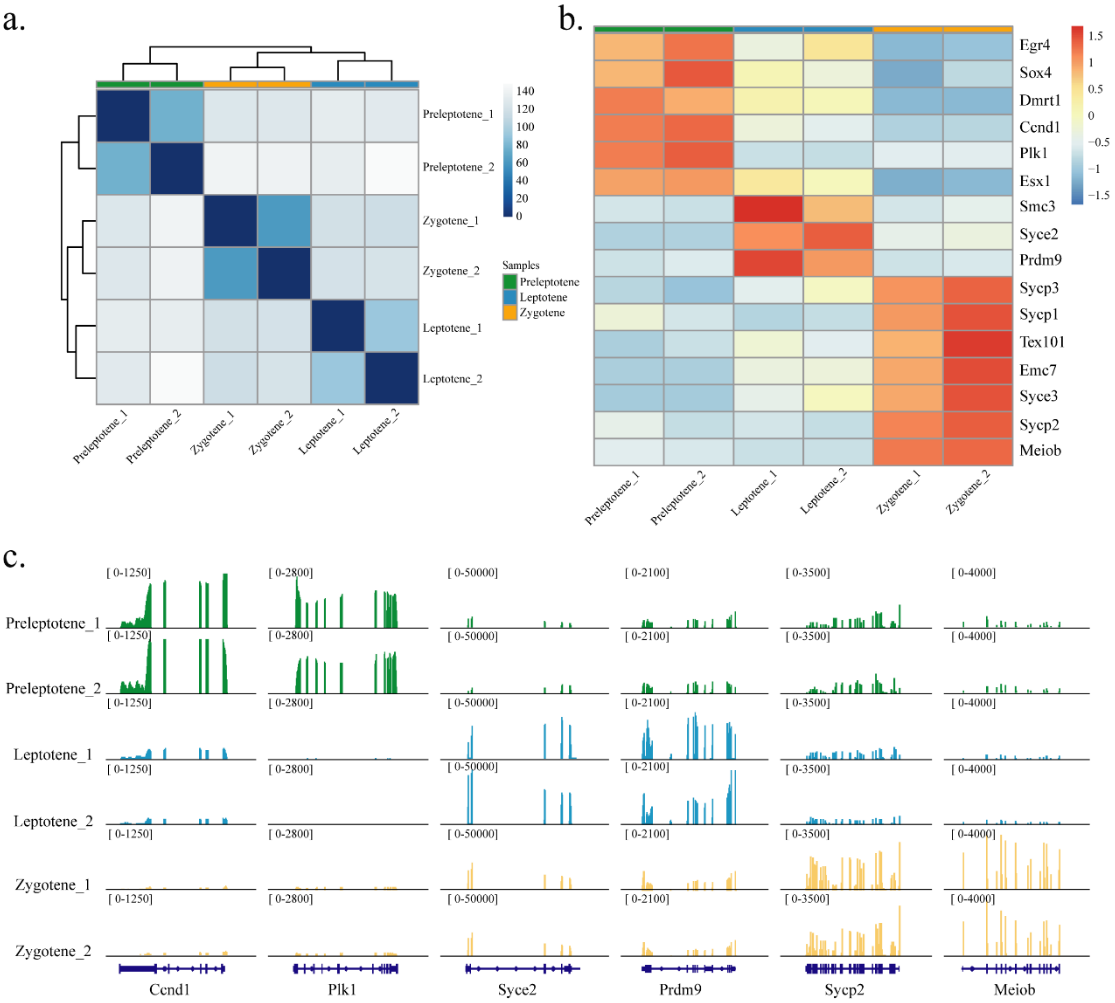

Supplementary Figure 3. Transcriptional signatures revealed by bulk RNA-Seq confirmed the identity of isolated spermatocytes

a, Bulk RNA-Seq was performed on two biological replicates of FACS-isolated preleptotene, leptotene, and zygotene stage spermatocytes. Sample distances matrix indicates the high reproducibility of bulk RNA-Seq datasets.

b, Normalized expression counts for a set of marker genes of preleptotene, leptotene, and zygotene spermatocytes derived from a previous single-cell RNA-Seq study[42].

c, RNA-Seq tracks for representative marker genes associated with preleptotene, leptotene, and zygotene stages.

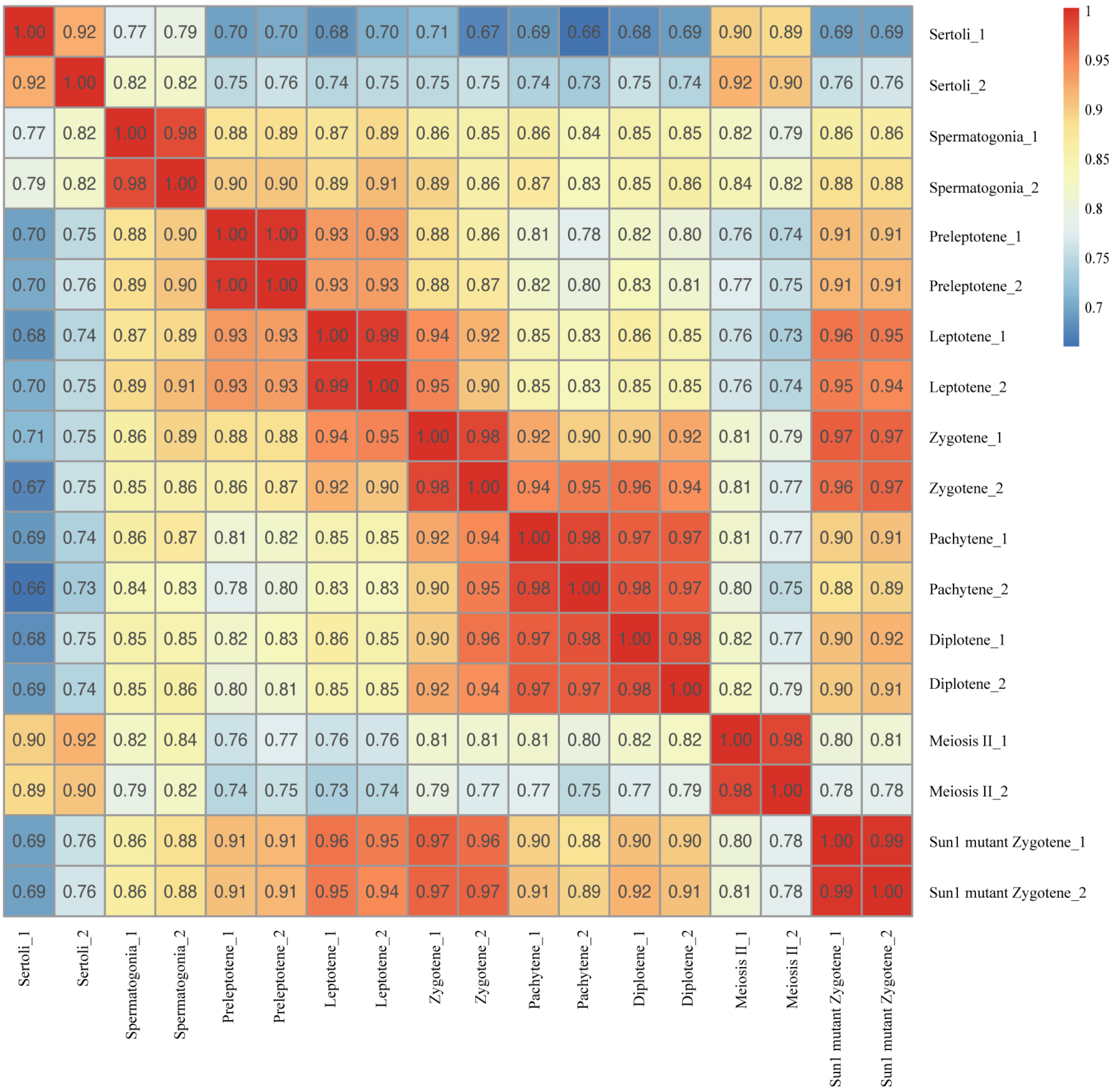

Supplementary Figure 4. Assessment of reproducibility of Hi-C replicates

Heatmap shows correlations between pairwise combinations of Hi-C datasets generated in this study. Pearson correlation coefficients were calculated using balanced chromatin interactions binned at 500 kb and the HiCRep package in R. Overall, the two biological replicates for each stage are highly correlated with each other, indicating a high degree of reproducibility.

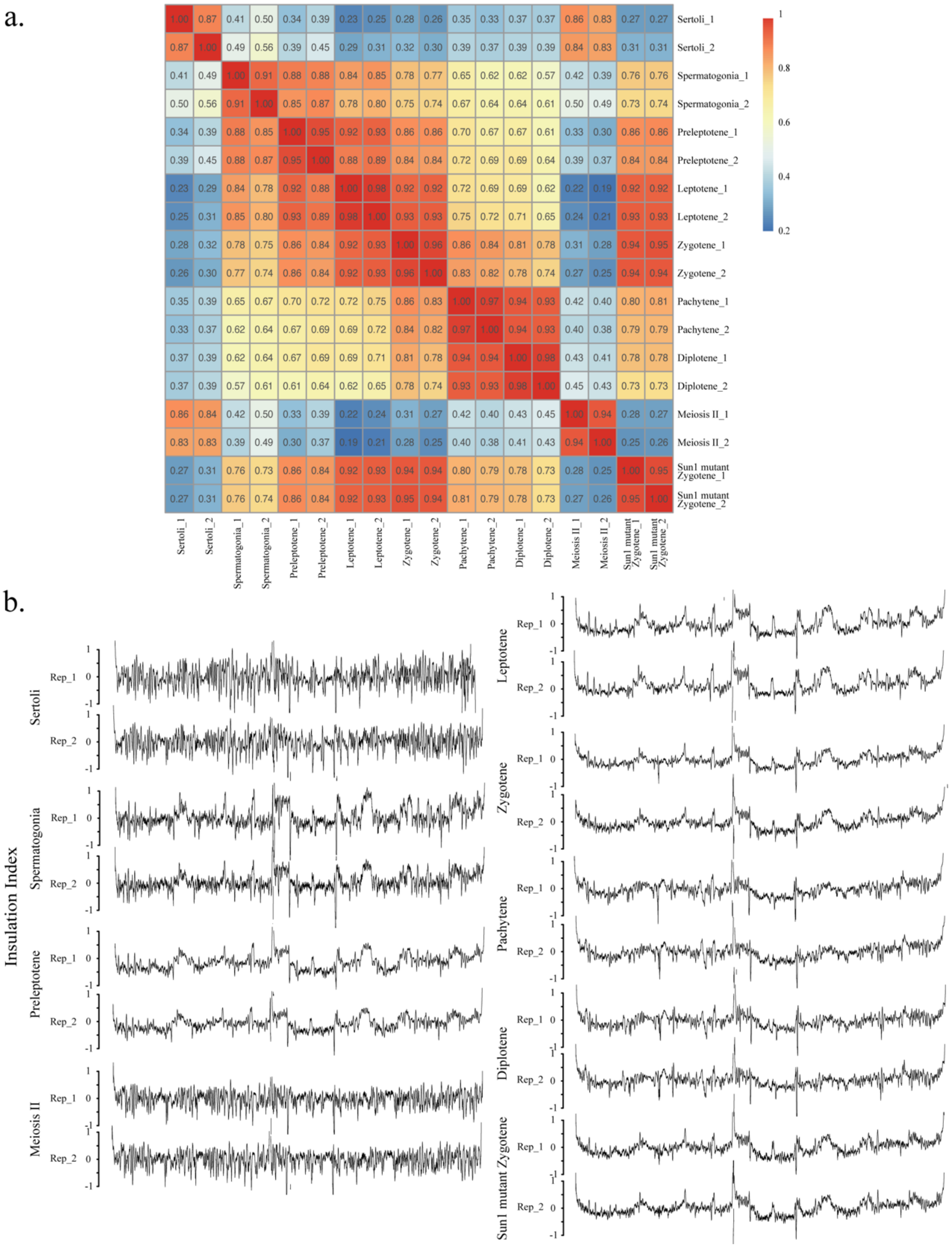

Supplementary Figure 5. Reproducibility of insulation profiles for Hi-C replicates

a, Pearson correlation coefficients were calculated using insulation profiles at 10 kb resolution for the pairwise combination of Hi-C datasets. Overall, the two biological replicates for each stage are highly correlated with each other, indicating a high degree of reproducibility.

b, Insulation profiles at 10 kb resolution for Chr1 in each Hi-C dataset. The local

minima on the insulation profiles denote locations of high insulation ability, which correspond to TAD boundaries.

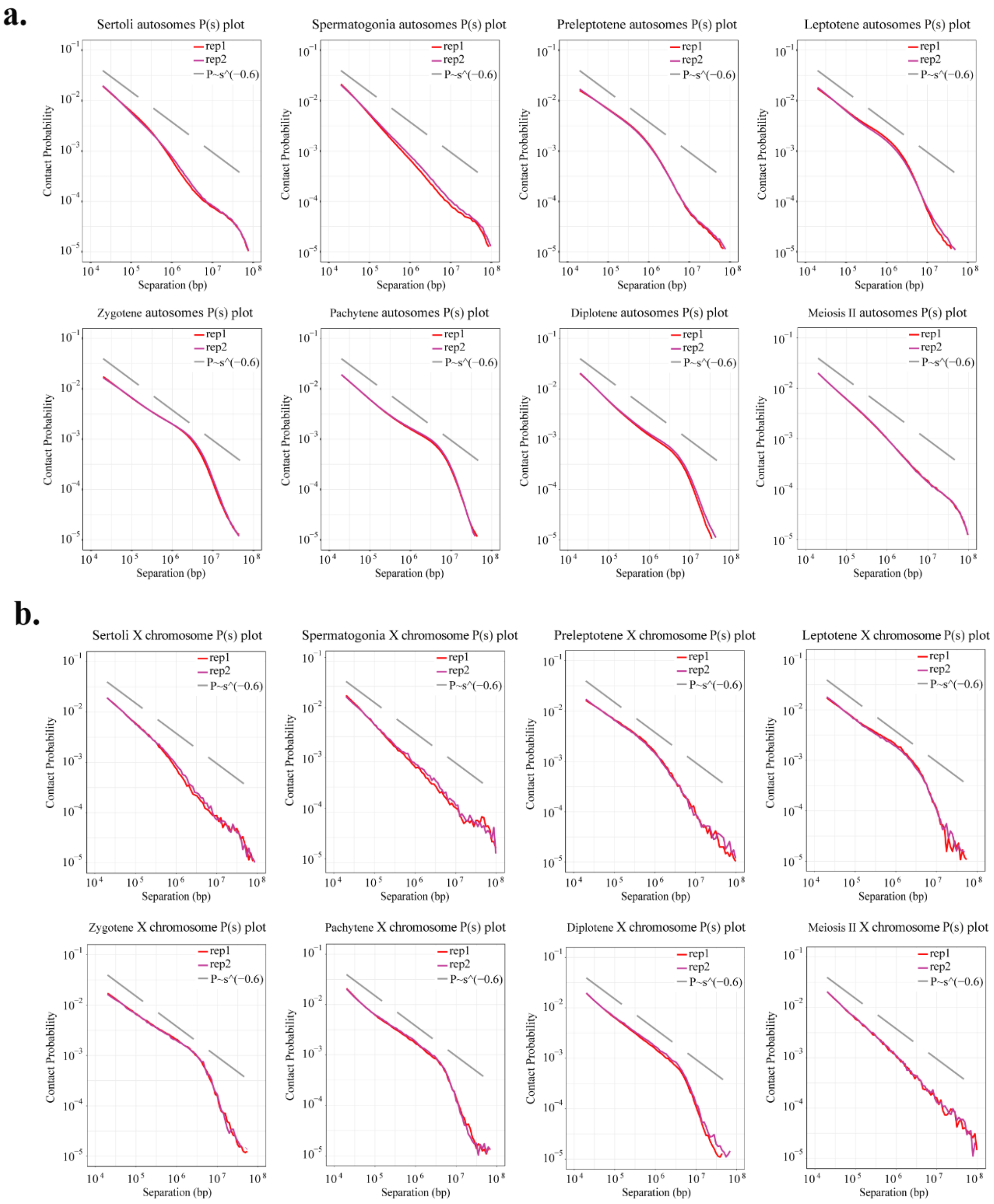

Supplementary Figure 6. Reproducibility of P(s) curves for Hi-C replicates

a, b, P(s) curves indicate relationships between chromatin contact probability and genomic distances for chromatin interactions on autosomes (a) and the X chromosome (b) in two Hi-C replicates for each of eight different cell stages. The dotted line corresponding to $P(s) \sim s^{-0.6}$ is shown as a reference.

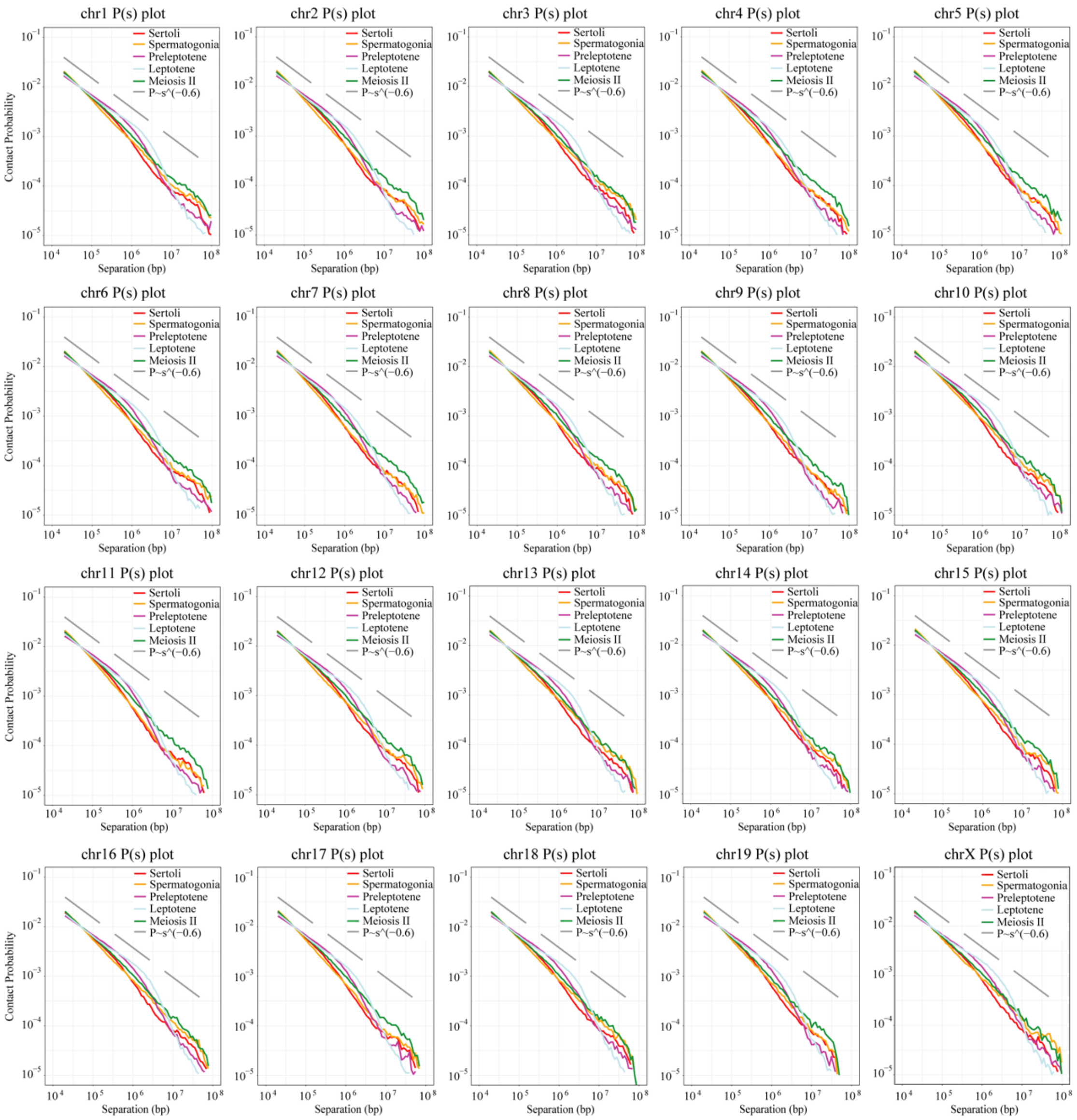

Supplementary Figure 7. P(s) curves before and after meiotic prophase I for the individual autosome

P(s) curves indicate relationships between chromatin contact probability and genomic distances for chromatin interactions on individual autosomes (Chr1-19) and the X chromosome in Sertoli, spermatogonia, preleptotene, leptotene, and meiosis II cells.

The dotted line corresponding to P(s) ~ $s^{-0.6}$ is shown as a reference.

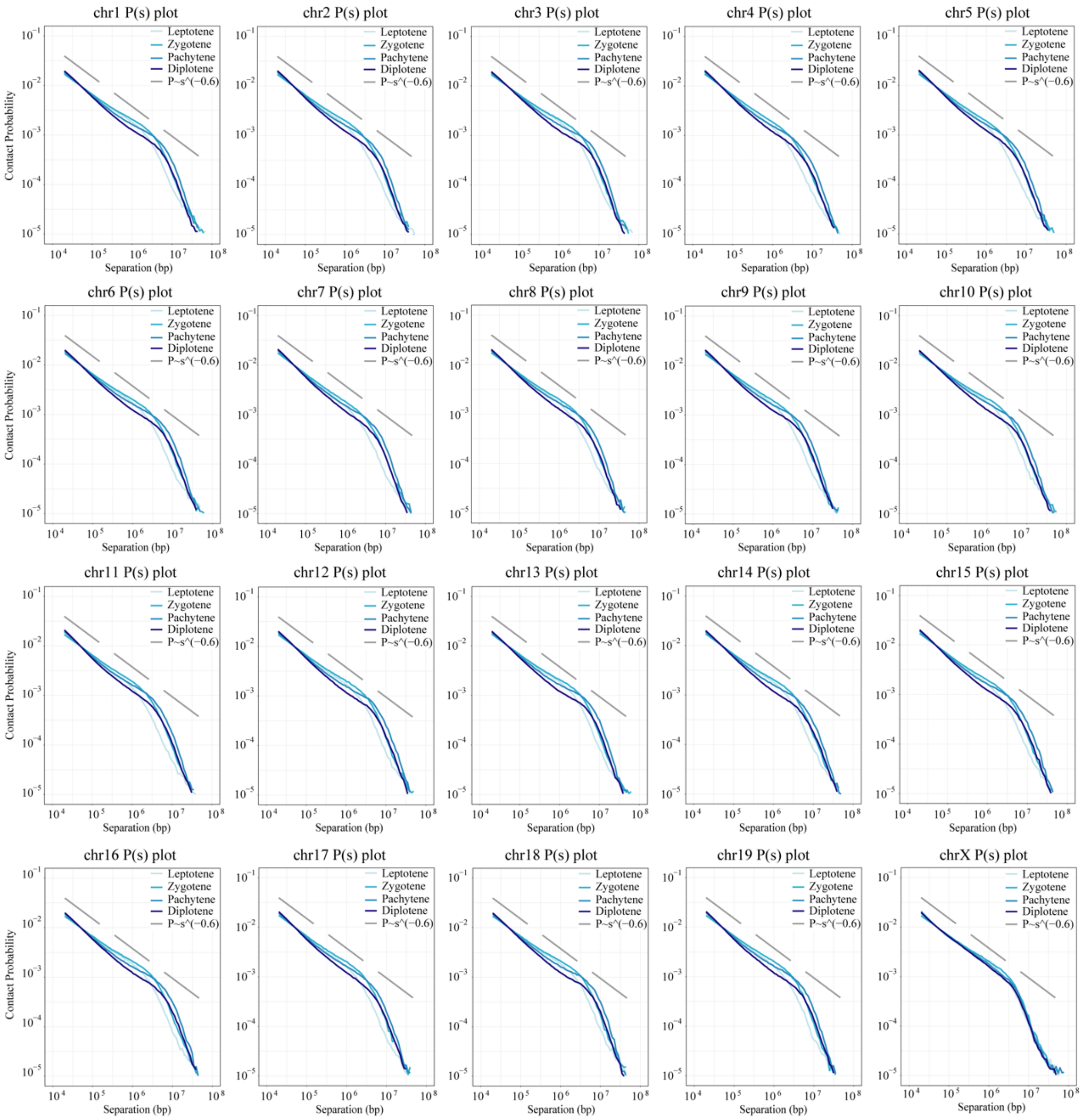

Supplementary Figure 8. P(s) curves during meiotic prophase I progression for the individual autosome

P(s) curves indicate relationships between chromatin contact probability and genomic distances for chromatin interactions on individual autosomes (Chr1-19) and the X chromosome in leptotene, zygotene, pachytene, and diplotene cells. The dotted line corresponding to P(s) ~ $s^{-0.6}$ is shown as a reference.

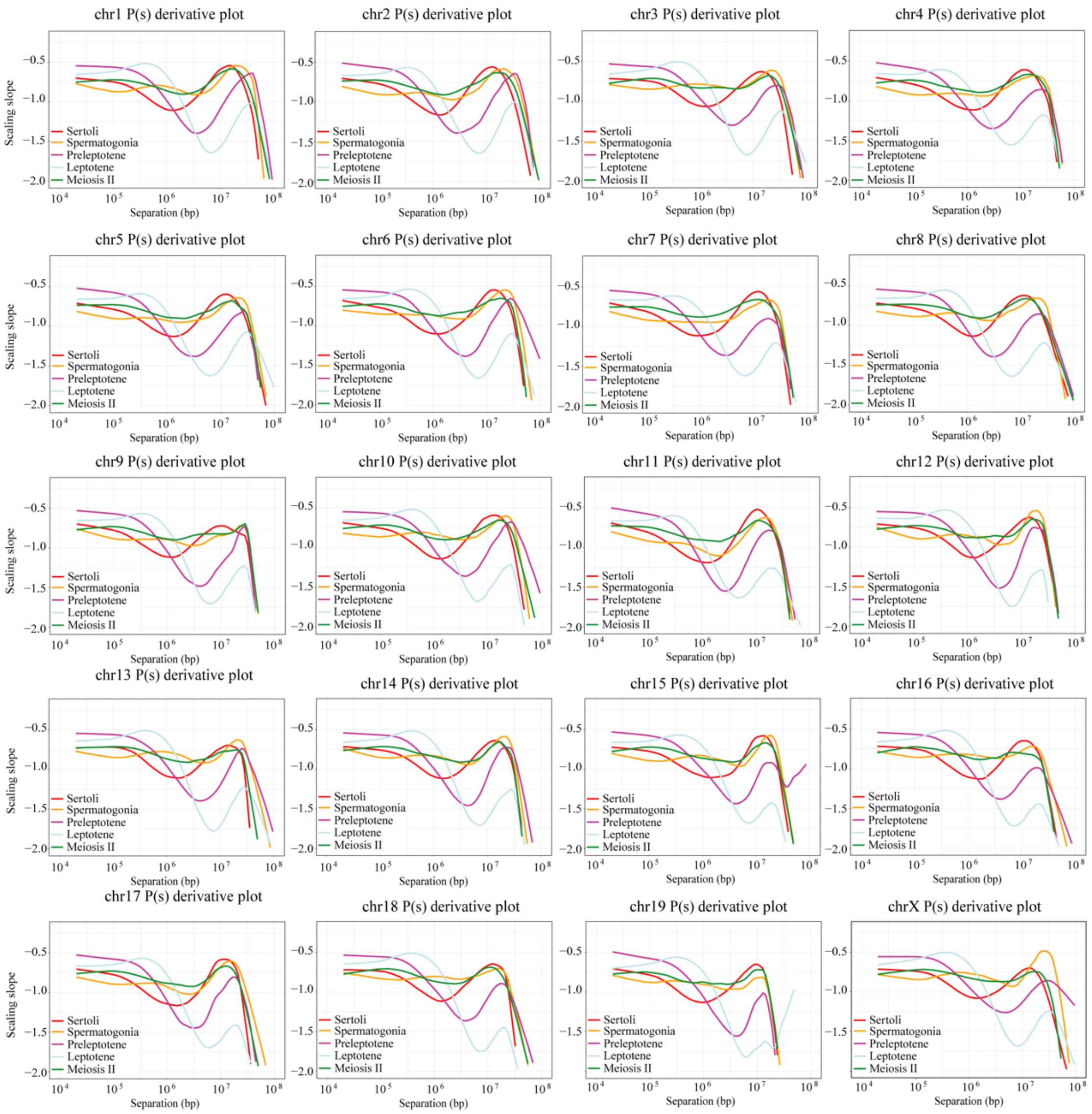

Supplementary Figure 9. Changes of chromatin loop size before and after meiotic prophase I for the individual autosome

Slopes of P(s) curves in Supplementary Figure 7 at different genomic distances are used to infer the average chromatin loop size on individual autosomes (Chr1-19) and the X chromosome in Sertoli, spermatogonia, preleptotene, leptotene, and meiosis II cells.

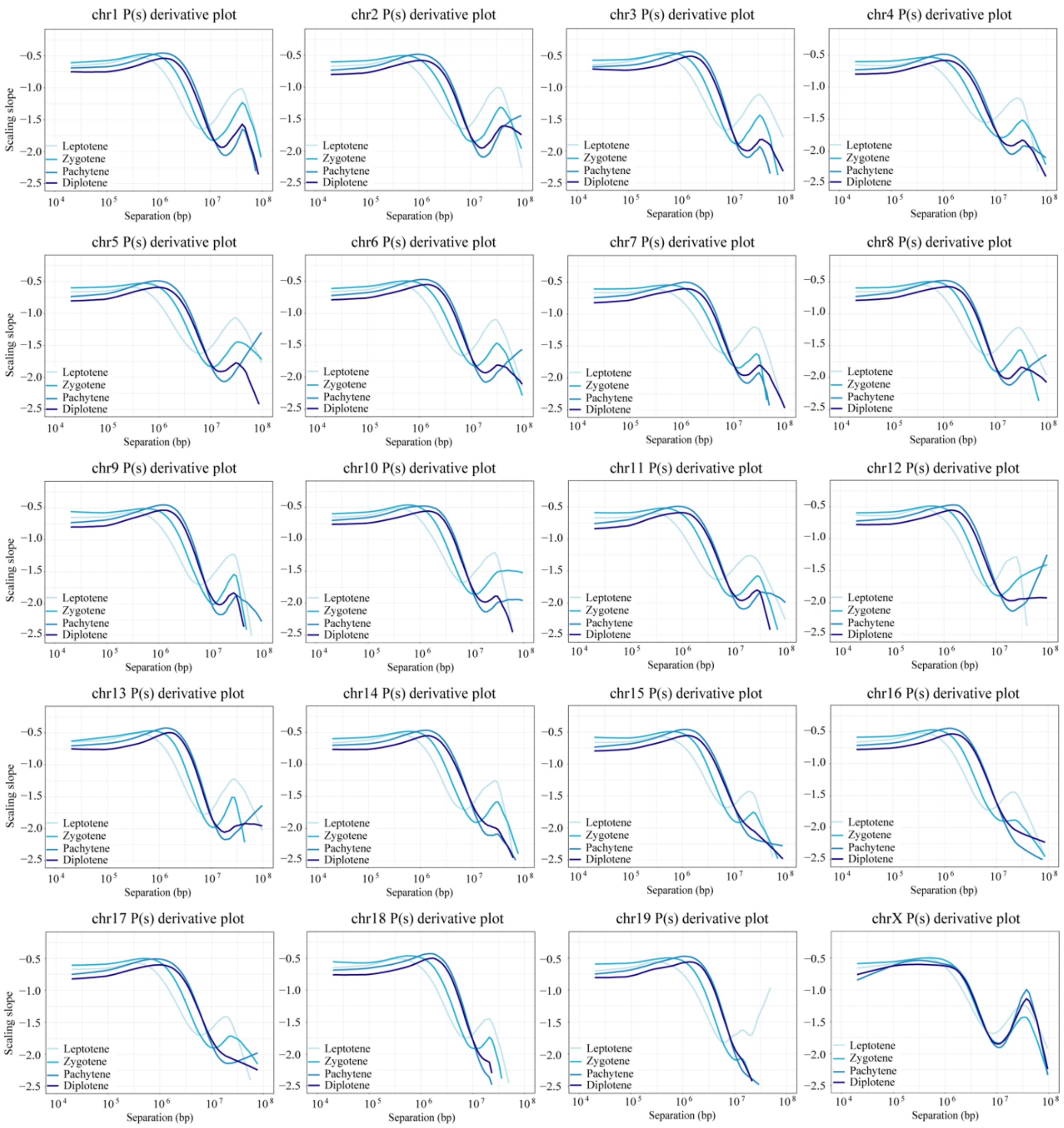

Supplementary Figure 10. Changes of chromatin loop size during meiotic prophase I progression for the individual autosome

Slopes of P(s) curves in Supplementary Figure 8 at different genomic distances are used to infer the average chromatin loop size on individual autosomes (Chr1-19) and the X chromosome in leptotene, zygotene, pachytene, and diplotene spermatocytes. Chromatin loop sizes progressively increase from the leptotene to the diplotene stage on each autosome.

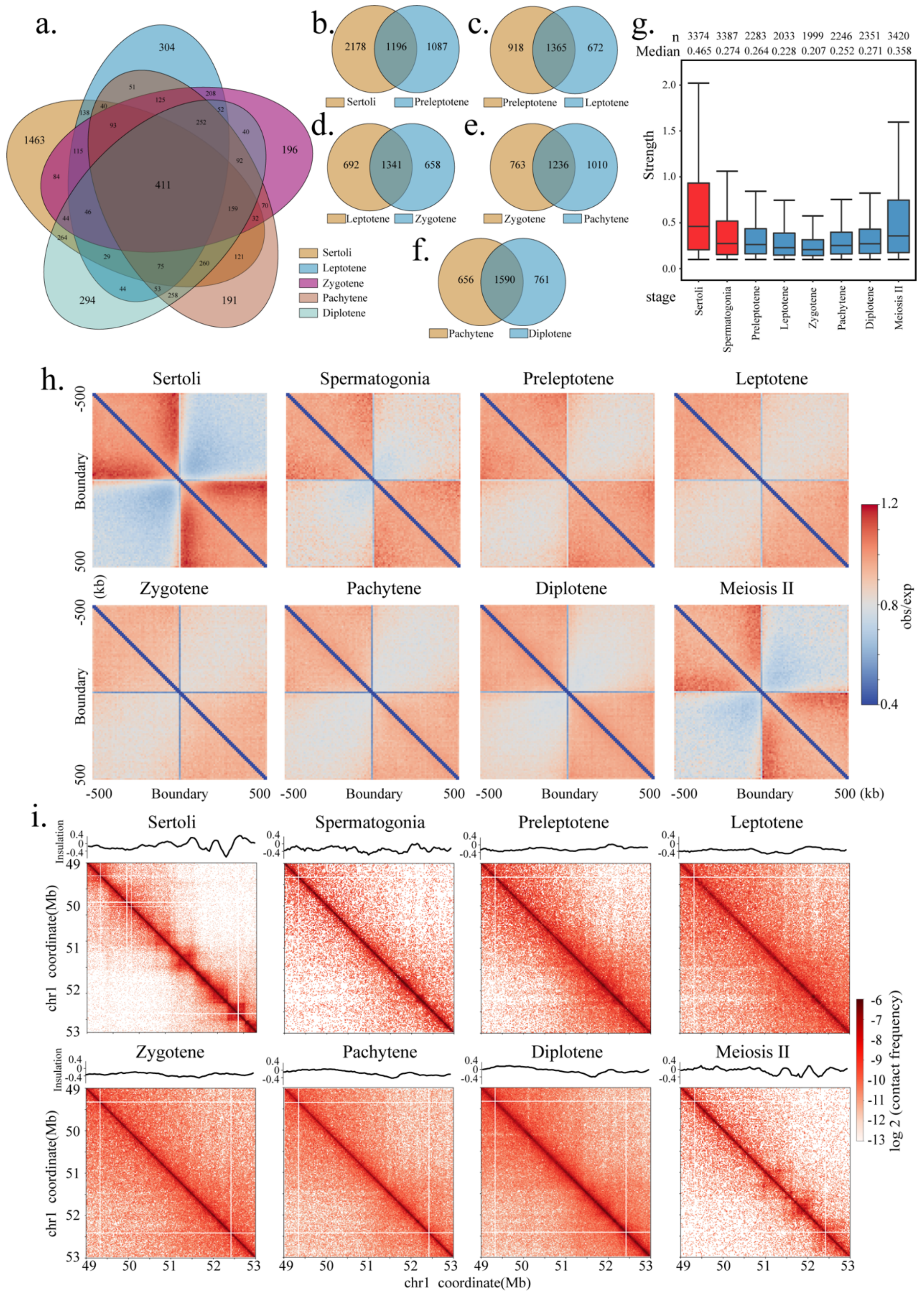

Supplementary Figure 11. Weakening of TAD organization during meiotic prophase I

a, Venn diagram depicts the overlap between TAD boundaries identified in Sertoli cells and the leptotene, zygotene, pachytene, and diplotene stage spermatocytes. Only a small

fraction of TAD boundaries are conserved in all cell types.

b-f, Venn diagrams depict the overlap of TAD boundaries between Sertoli cells and preleptotene spermatocytes (b), and the overlap of TAD boundaries between successive meiotic prophase I substages (c-f).

g, Box plots quantify the strength of TAD boundaries. Note that the TAD boundary Strength score is a different metric from the insulation indices. Higher values in TAD boundary Strength scores indicate stronger TAD boundaries. Stages before and after meiosis entry are labeled in red and blue. The upper and lower bounds of boxes represent the third and the first quartiles of TAD boundary strength, respectively. Centre bars represent the median of TAD boundary strength. The upper whisker extends from the hinge to the largest value no further than 1.5 × IQR (inter-quartile range) from the hinge, and the lower whisker from the hinge to the lowest value within 1.5 * IQR of the hinge. The values beyond the whiskers are not shown in the boxplots. n, the total number of TAD boundaries identified in each stage. The n values are indicated on the top of the boxplot.

h, Pileup heatmaps of 500 kb genomic regions flanking each TAD boundary in different cell types reveal weakening of TAD boundaries during meiotic prophase I. The observed Hi-C interaction frequencies are normalized using expected interaction frequencies at each genomic distance (Obs/Exp). The blue crosses observed at the center of the heatmaps arise from the large number of TAD boundaries located at genomic bins lacking uniquely mapped reads. Bin size, 10 kb.

i, Hi-C heatmaps of a representative region on chr1 (48– 53 Mb) throughout mouse spermatogenesis. The corresponding insulation profiles are shown on top of the heatmaps. The TAD boundaries are evident in Sertoli cells but largely diminished in preleptotene and all subsequent meiotic stages. Bin size, 20 kb.

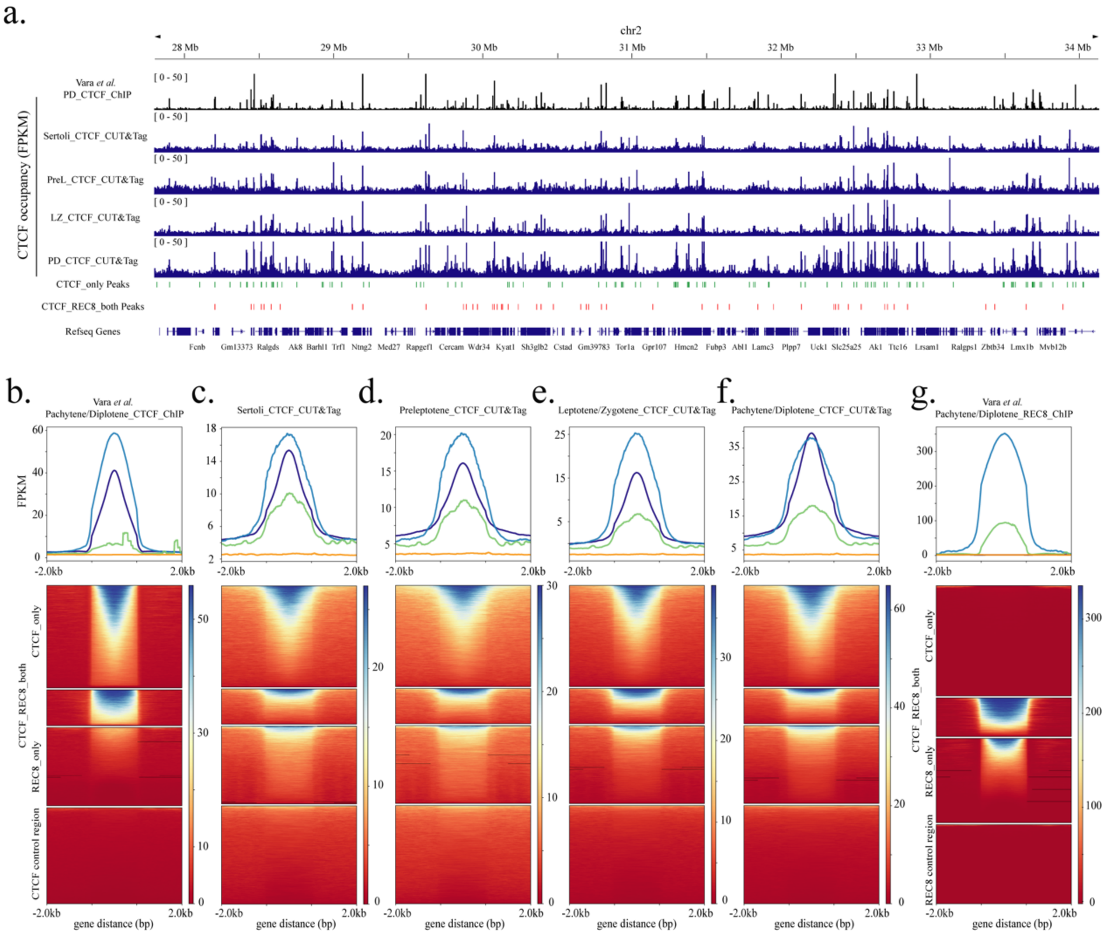

Supplementary Figure 12. Persistent CTCF binding throughout meiotic prophase I

a, ChIP-seq and CUT&Tag profiles show CTCF peaks and distribution in a representative genomic region (Chr2: 28-34 Mb). The CUT&Tag profiles in Sertoli, preleptotene, leptotene/zygotene, and pachytene/diplotene cells exhibit a similar distribution of CTCF as the pachytene/ diplotene stage ChIP-Seq data from Vara *et al.*[34]. Green and Red bars indicate the peaks that are only occupied by CTCF, or the peaks that are occupied by both CTCF and REC8. Positions of peaks in both categories are largely preserved in different meiotic stages, suggesting that CTCF remains bound to chromosomes during meiosis.

b-f, The CTCF occupancy patterns at the 6118 CTCF/ REC8 co-occupied peaks, the 17613 CTCF-only, and the 13561 REC8-only peaks occupancy patterns in different ChIP-seq and CUT&Tag datasets are depicted using averaged line graph and heatmap. Each row in the heatmap represents the CTCF occupancy at one peak and the 2 kb

genomic regions flanking the peak. A set of genomic regions generated by randomizing the positions of the 17613 CTCF-only peaks were used as control. Within each ChIP-seq and CUT&Tag profile, the CTCF occupancy at the CTCF/ REC8 co-occupied peaks is at a similar level to or higher than the CTCF occupancy at the CTCF-only peaks.

g, The REC8 occupancy at different types of peaks in pachytene/ diplotene stage ChIP-Seq data are depicted as in b-f. Control regions were generated by randomizing the positions of the 13561 REC8-only peaks. Both the line graph and the heatmap indicate higher REC8 occupancy at the CTCF/ REC8 co-occupied peaks.

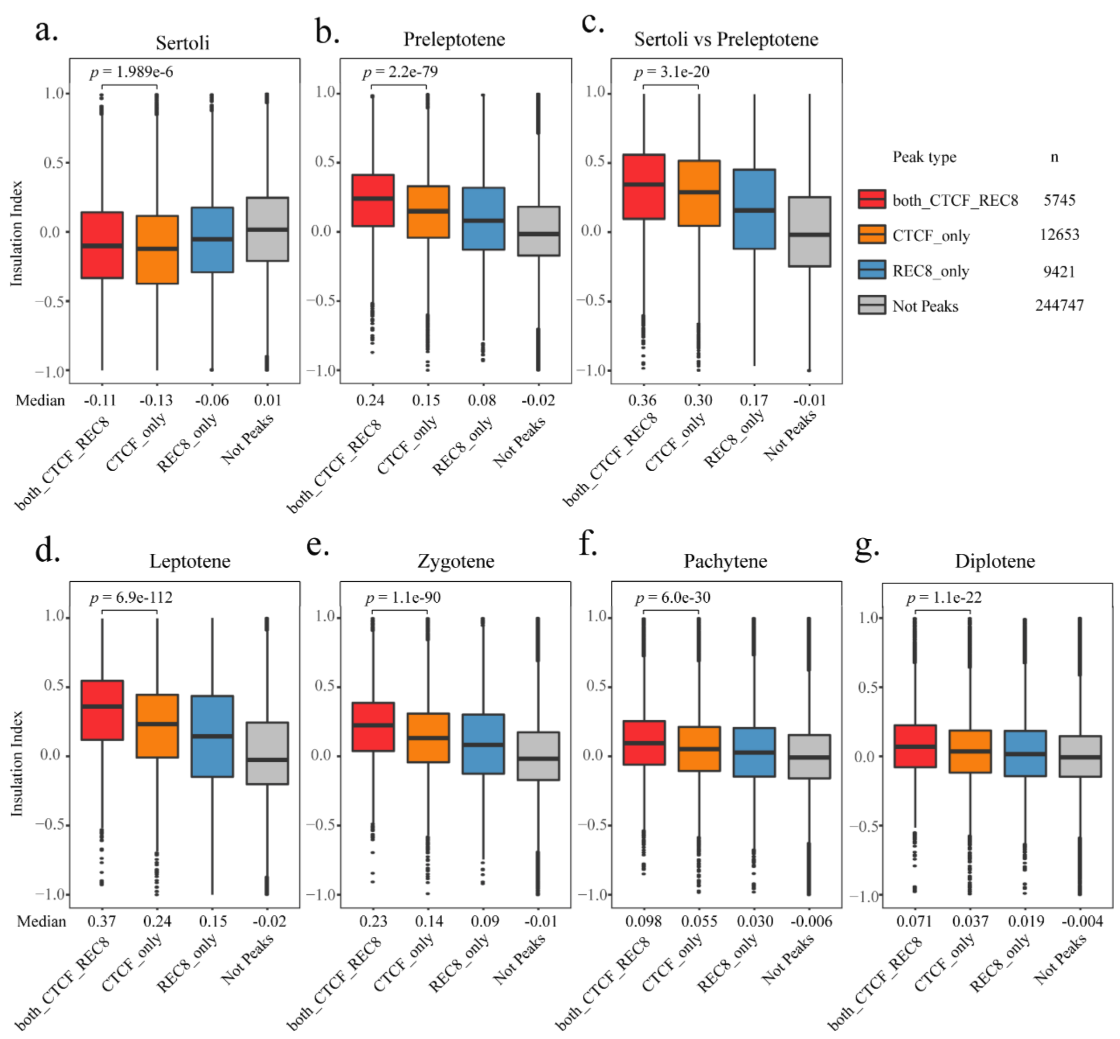

Supplementary Figure 13. Loss of insulation abilities at the CTCF-binding sites during meiotic prophase I.

Box plots quantify the insulation scores at 10 kb genomic intervals coinciding with the CTCF/ REC8 co-occupied peaks, CTCF-only peaks, REC8-only peaks, and the genomic intervals not coinciding with any peaks at different stages. The number of genomic intervals in each category is indicated in the keys. Box limits, upper and lower quartiles. Centre bars, median. Whiskers, 1.5x interquartile range. n, the total number of the CTCF/ REC8 co-occupied peaks (n = 5745), CTCF-only peaks (n = 12653), REC8-only peaks (n = 9421), and the genomic intervals not coinciding with any peaks (n = 244747).

a, The genomic intervals coinciding with the CTCF/ REC8 co-occupied peaks (Median = -0.11) and CTCF-only peaks (Median = -0.13) exhibit low insulation scores, indicating strong insulation abilities at these locations, consistent with the function of

CTCF to impede chromatin loop extrusion during interphase and form TAD boundaries. The insulation scores at the CTCF/ REC8 co-occupied peaks are higher than the CTCF-only peaks (p = 1.989 x 10^{-6}, two-tailed Mann-Whitney U-test). Notably, the genomic intervals coinciding with the REC8-only peaks also exhibit lower insulation scores than the genomic background, which could be attributed to the CTCF binding detected by CUT&Tag at some of these regions (Supplementary Figure 12c).

b, In preleptotene, the genomic intervals associated with all three categories of peaks exhibited significantly higher insulation scores compared to the non-peak regions. Since the insulation scores measure the aggregated interactions occurring across a specific genomic interval. The high, positive values at these locations suggest that rather than forming boundaries, these locations become sites of high “conductivity”. The CTCF/ REC8 co-occupied peaks exhibit the highest insulation scores (Median = 0.24) among all groups of sites. Notably, the insulation scores at the CTCF/ REC8 co-occupied peaks are significantly higher than those at the CTCF-only peaks (p = 2.2 x 10^{-79}, two-tailed Mann-Whitney U-test).

c, Boxplots quantify the changes of insulation scores at each genomic interval between preleptotene and Sertoli cells (preleptotene - Sertoli). The genomic regions associated with all three types of peaks exhibit marked increases in insulation scores, among which the regions associated with the CTCF/ REC8 co-occupied peaks exhibit the highest insulation increases (Median = 0.36) than the other groups, including the CTCF-only peaks (p = 3.1 x 10^{-20}, two-tailed Mann-Whitney U-test). The fact that the REC8 occupancy correlates with the increases in insulation scores implies that tethering to the chromosome axis may impair the insulator function of CTCF.

d-g, The CTCF/ REC8 co-occupied peaks exhibit the highest insulation scores than all other groups of sites, including the CTCF-only peaks, throughout the entire meiosis (p = 6.9 x 10^{-112} for leptotene, p = 1.1 x 10^{-90} for zygotene, p = 6.0 x 10^{-30} for pachytene, p = 1.1 x 10^{-22} for diplotene, two-tailed Mann-Whitney U-test). However, the insulation scores at peaks gradually decrease in later meiotic Prophase I, suggesting the insulation abilities may be restored at some sites.

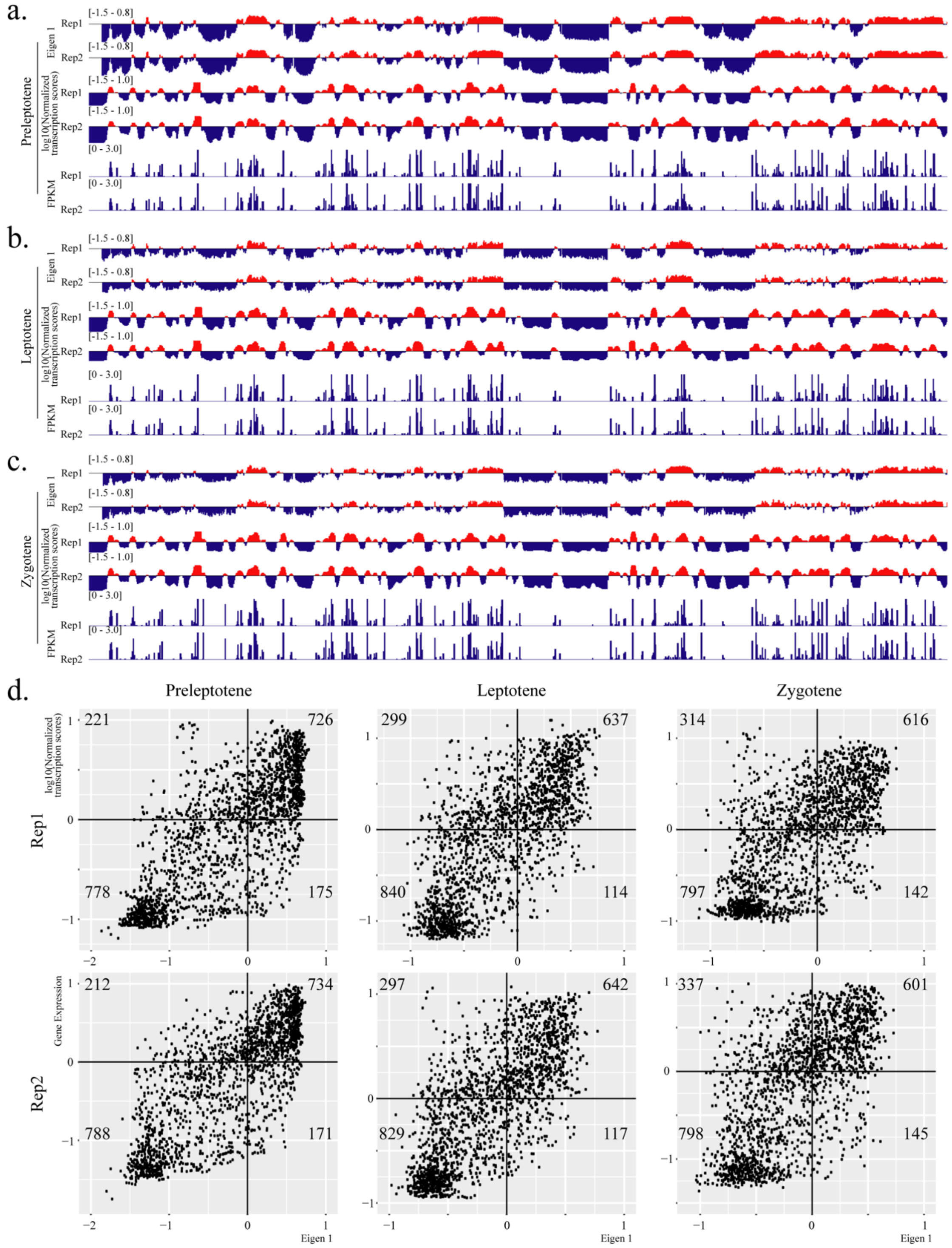

Supplementary Figure 14. Compartment identities correlate with transcriptional activities during meiosis

a-c, Tracks of Eigen1 values, log10 (Normalized transcription scores), and the RNA-Seq reads on the entire Chr1 depict the correlation between A/B compartment identity and transcription activities in preleptotene (a), leptotene (b), and zygotene (c). For each stage, Eigen1 values (top) are derived from the cool files for two Hi-C replicates at a

100 kb bin size, with red and blue colors indicating A and B compartment, respectively. RNA-Seq reads tracks (bottom) show the read coverage at 100 kb bin size in FPKM from two independent bulk RNA-Seq replicates. log10 (Normalized transcription activities) (middle) are generated by first smoothening the FPKM tracks using a 1% loess smoothing window, followed by normalizing the smoothened transcription scores using the median and performing a log10 transformation.

d, Scatter plots show correlations between the Eigen1 values from two independent Hi-C replicates and the log10 (Normalized transcription scores) from two independent RNA-Seq replicates for all 100 kb genomic bins on Chr1. The numbers of bins in each quadrant are indicated. Genomic bins of high and low transcriptional activities are primarily identified as A and B compartment, respectively, in all three stages analyzed here.

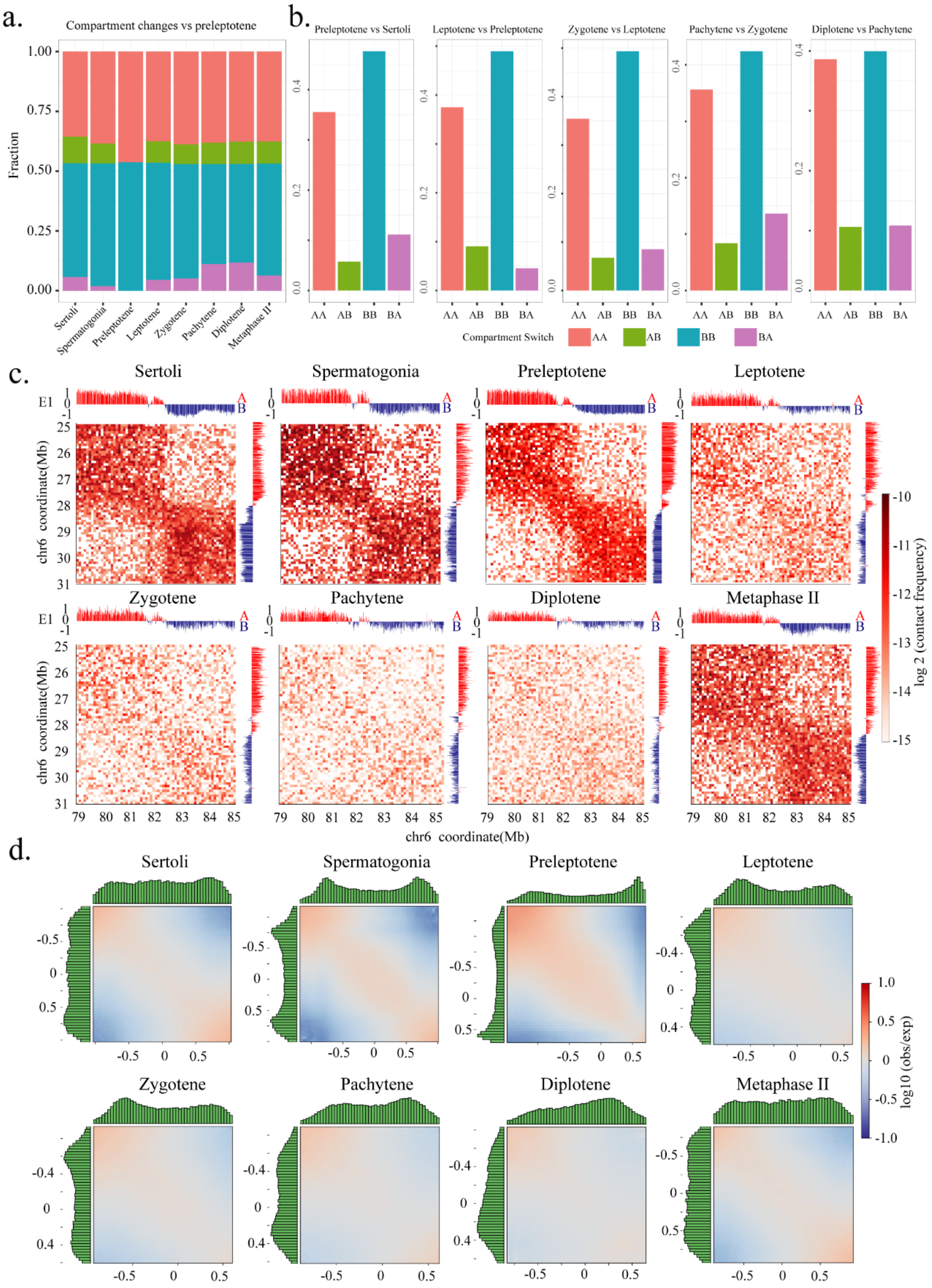

Supplementary Figure 15. A/B compartments are maintained but attenuated during meiotic prophase I

a, Stacked bar graph depicts the changes in A/ B compartment identity in every stage compared to the preleptotene stage. A/ B compartment identity for every 10 kb genomic bin is determined based on the Eigen1 value. AA, AB, BB, and BA represent

the 10 kb bin maintaining the A compartment identity, switching from A to B compartment, maintaining the B compartment identity, and switching from B to A compartment, respectively. The compartment identities are largely stable throughout the entire meiosis.

b, Bar graphs showing the compartment changes between Sertoli cells and preleptotene spermatocytes and between successive meiotic prophase I substages.

c, Hi-C heatmaps show the loss of the long-range association between two genomic regions on chr6 that are separated by more than 40 Mb (Chr6: 25 Mb- 31 Mb and Chr6: 79 Mb- 85 Mb) during meiosis. In Sertoli cells, the regions belonging to the same compartment preferentially associate with each other (upper-left corner: A-A association, bottom-right corner: B-B association), while not associating with regions belonging to the different compartment. The long-range association was prominent in preleptotene, but largely diminished in leptotene, zygotene, pachytene, and diplotene stages. Bin size, 100 kb.

d, Saddle plots quantify the overall level of genome compartmentalization in each stage. During meiosis, while the association between regions belonging to the same compartment decreases (upper-left corner: A-A association, bottom-right corner: B-B association), interactions between A and B compartment (upper-right and bottom-left corners) remain at a low level.

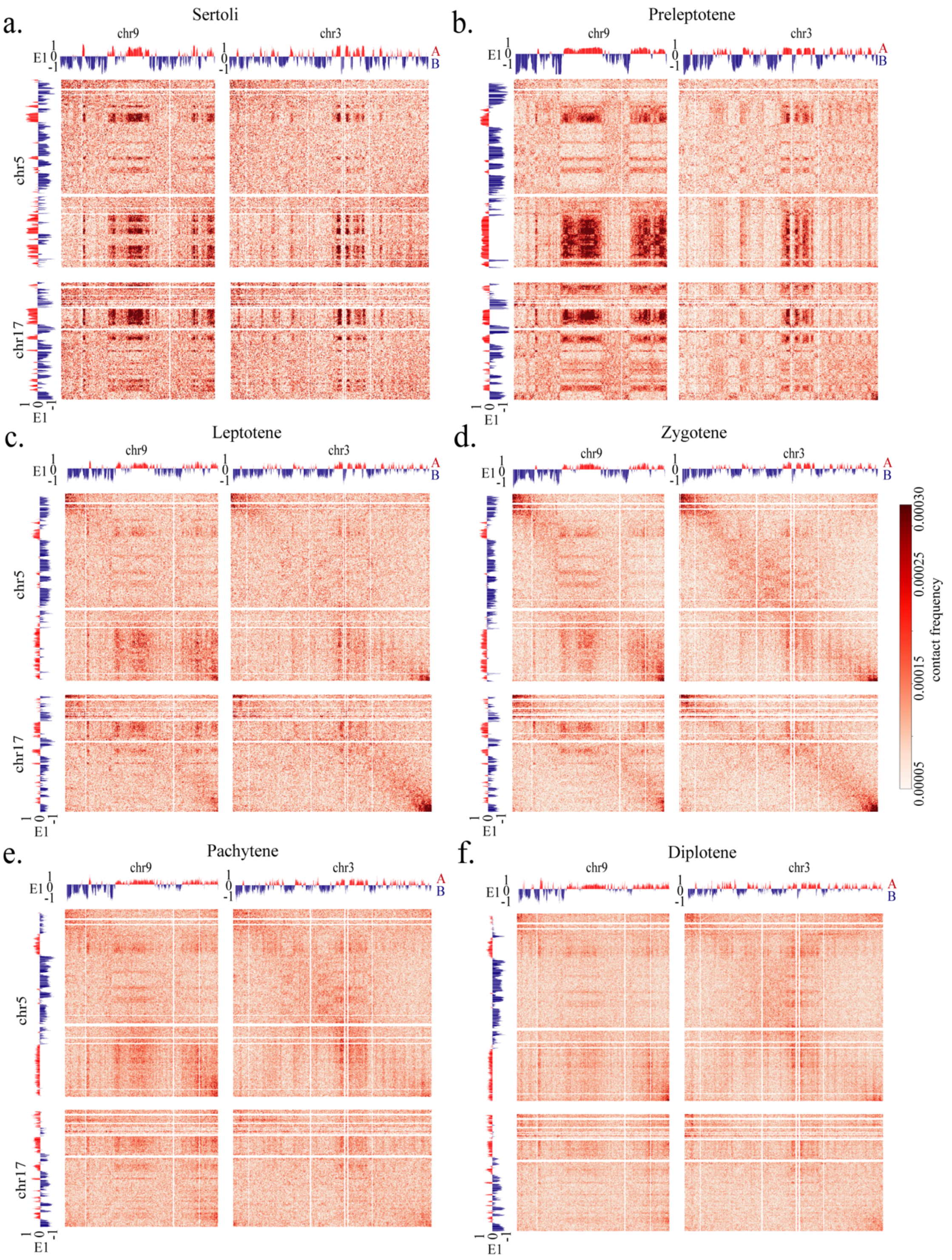

Supplementary Figure 16. Dynamic changes of inter-chromosomal interactions during meiotic prophase I

a-e, Inter-chromosomal interaction heatmaps between chromosomes 3 and 5, chromosomes 3 and 17, chromosome 5 and 9, and chromosomes 9 and17 in Sertoli (a), preleptotene (b), leptotene (c), zygotene (d), pachytene (e) and diplotene (f). Plots of eigenvector 1 values denoting A (red) and B (blue) compartments are shown on the top

and the left side of the heatmaps. Chromosome ends exhibit extensive association during leptotene and zygotene, but not other stages. Bin size, 500 kb.

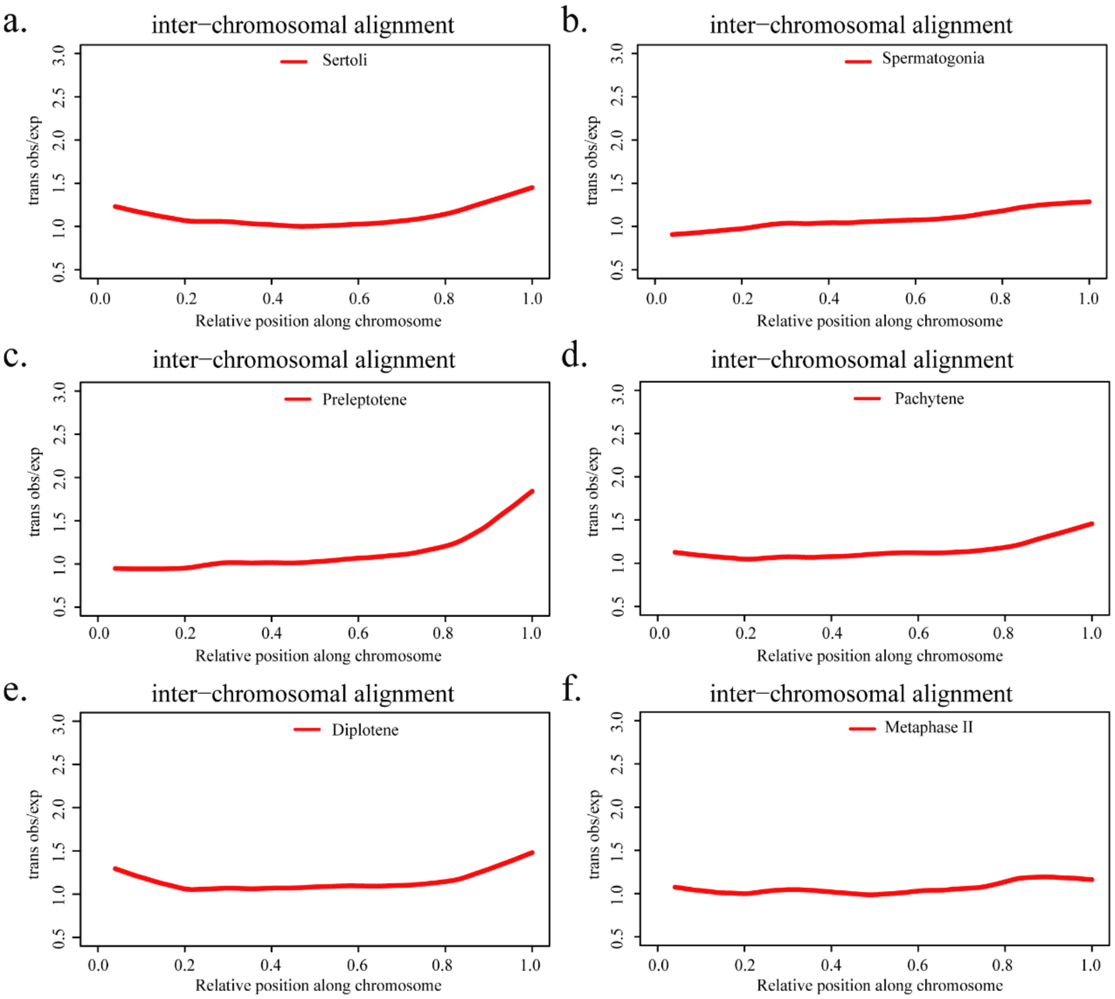

Supplementary Figure 17. Quantification of inter-chromosomal interactions relative to chromosome locations during meiotic prophase I

Plots showing trans observed/ expected interaction signals along the chromosome length in Sertoli (a), spermatogonia (b), preleptotene (c), pachytene (d), diplotene (e), and meiosis II (f).

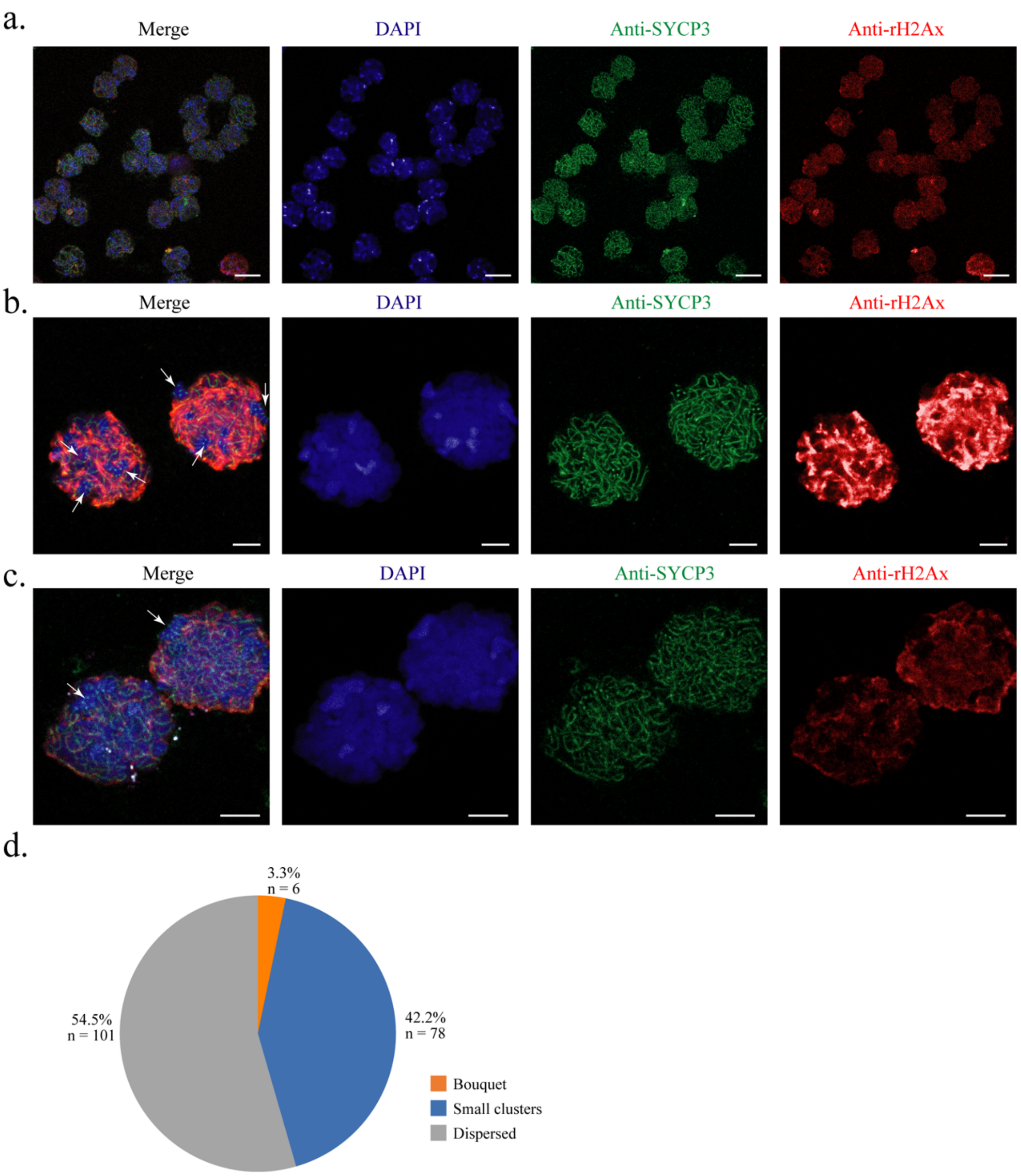

Supplementary Figure 18. Evaluating the clustering of chromosome ends in isolated zygotene spermatocytes using immunofluorescence.

a-c, Immunofluorescence was performed on zygotene spermatocytes that were not subject to hypotonic treatment to evaluate the chromosome conformation. Imaging was performed on cells isolated from three independent FACS sorting experiments.

a, Representative images showing zygotene spermatocytes stained with rH2AX (Red) and SYCP3 (green) antibodies and counterstained with DAPI (blue). Cells in these images were not subject to hypotonic treatment. Scale bar, 10 μm.

b, Two nuclei in which chromosome ends are organized into small clusters (arrows).

Each of the clusters contains 5-6 chromosomes. The clusters are distributed at different locations within the nuclei. Scale bar, 5 µm.

c, Two nuclei in which chromosome ends are organized into large clusters consisting of >10 chromosomes (arrows) and adopt a polarized localization, indicating bouquet conformation. Scale bar, 5 µm.

d, Quantification of the cells exhibiting bouquet conformation in isolated zygotene cells from three independent experiments. In a total of 185 cells examined, only 6 cells (3.3%) exhibit the bouquet conformation, and 78 cells (42.2%) exhibit multiple small clusters of chromosome ends.

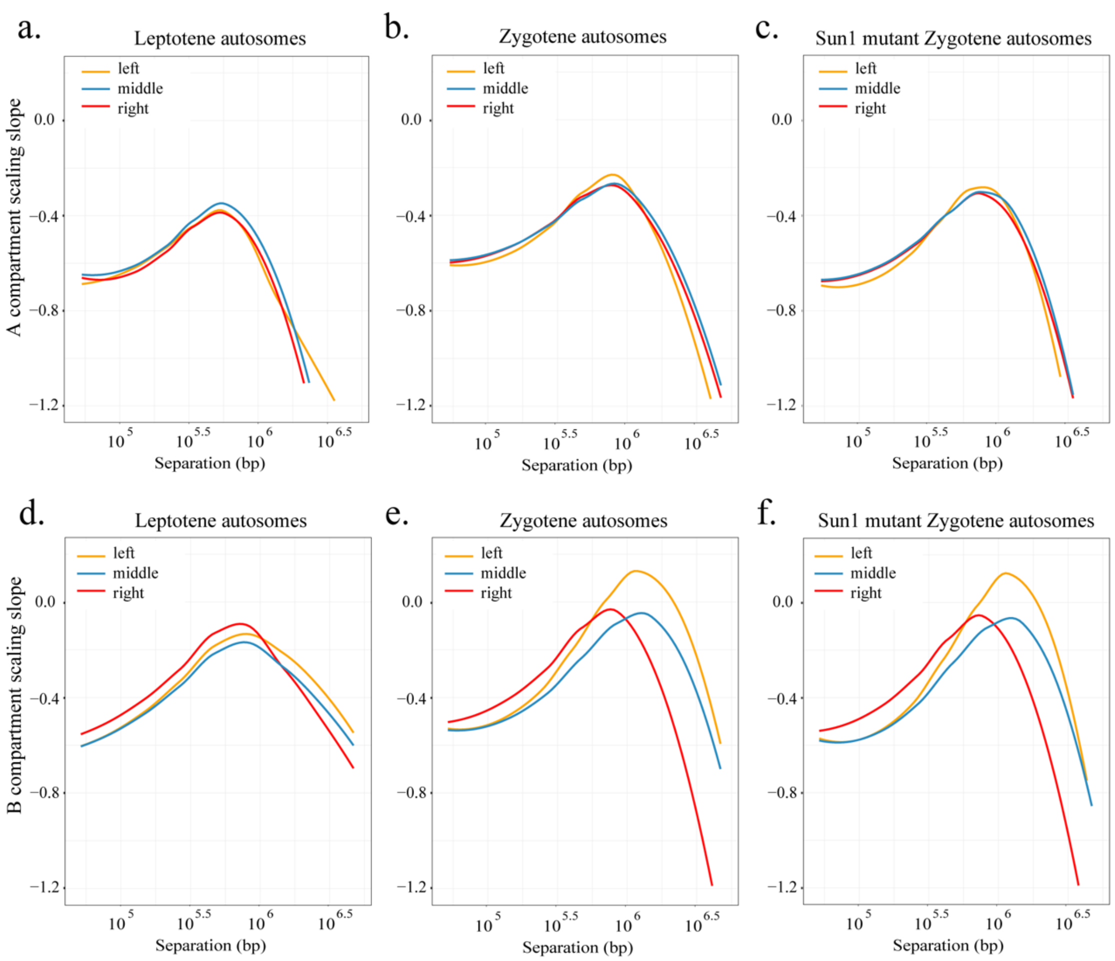

Supplementary Figure 19. Average chromatin loop sizes at chromosome ends and centers

a-f, Plots showing the relationships between the slopes of P(s) and genomic separation for genomic regions belonging to A or B compartment and located at different positions along chromosomes, with left, right, and middle correspond to genomic regions locating within 20% chromosome length from centromere proximal end, genomic regions locating within 20% chromosome length from centromere distal end and genomic regions locating in the middle 60% of chromosomes, respectively. Peak locations on the plots indicate average loop size.

a, b, For wild-type leptotene and zygotene spermatocytes, the A compartment regions located at different chromosome positions exhibit no difference in average chromatin loop sizes.

c, d, For wild-type leptotene and zygotene spermatocytes, the B compartment regions located near centromere distal ends exhibit smaller chromatin loop sizes than the rest

of chromosomes.

e, f, Sun1$^{W151R/W151R}$ mutant zygotene spermatocytes exhibit identical chromatin loop sizes as wild-type zygotene spermatocytes, suggesting the loss of inter-chromosomal alignment at chromosome ends in the mutant is not linked to modulation of chromatin loop sizes.

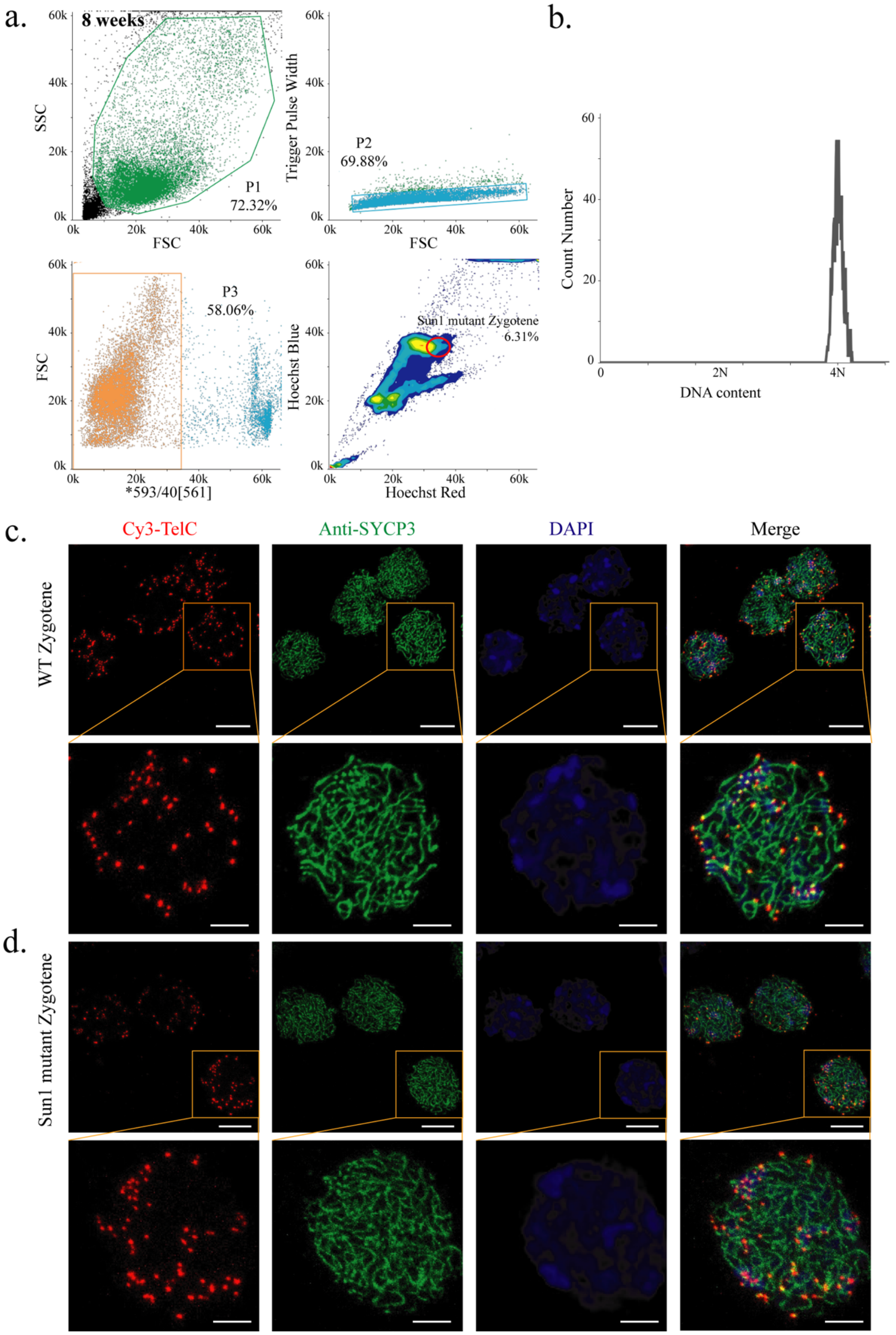

Supplementary Figure 20. SUN1 W151R mutation does not completely abolish the nuclear peripheral localization of telomeres

a, The FACS data panels show the sequential gating strategy for isolating

Sun1$^{W151R/W151R}$ mutant zygotene spermatocytes, as described in Supplementary Figure 1.

b, A ploidy profile for Sun1$^{W151R/W151R}$ mutant zygotene spermatocytes.

c-d, Maximum intensity projections of Z-stacks of wild-type zygotene (c) and Sun1$^{W151R/W151R}$ mutant zygotene (d) spermatocytes show that the telomeres are still largely anchored to the INM in the mutant. Fluorescence in situ hybridization (FISH) signals of the telomere probe are shown in red. Antibody staining signals of meiosis marker SYCP3 are shown in green. DAPI-stained DNA is shown in blue. For both c and d, scale bars of the upper row and lower row are 10 μm and 4 μm, respectively. Similar patterns were observed on cells from three biologically independent FACS isolated samples.

Table S1. Viability and Purity of isolated Spermatocytes for Hi-C

Stage	Batch	Age of animals	Number of animals	Amount of cells(k)	Viability	Amount for spreading(k)	Amount for Hi-C(k)	Microscopy counts of each stage								Total amount each batch(k)	Batch Viability	Batch Purity
								Preleptotene	Leptotene	Zygotene	Pachytene	Diplotene	No Signal	Total	Purity			
Preleptotene	Batch 1	12 days	5	490.00	88%	20.00	470.00	73	20	0	0	0	0	93	78.49%	1137.50	90.35%	91.11%
		10 days	8	687.50	92%	20.00	667.50	39	0	0	0	0	0	39	100.00%			
	Batch 2	10 days	5	399.00	85%	15.00	384.00	28	0	0	0	0	0	28	100.00%	959.72	89.31%	96.58%
		9 days	5	96.90	85%	20.02	76.88	58	0	0	0	0	0	58	100.00%			
		10 days	5	89.70	93%	20.00	69.70	82	4	0	0	0	0	86	95.35%			
		10 days	6	81.00	90%	20.00	61.00	27	3	0	0	0	0	30	90.00%			
		10 days	6	62.00	93%	20.00	42.00	89	9	0	0	0	0	98	90.82%			
		10 days	5	54.24	86%	20.00	34.24	58	1	0	0	0	0	59	98.31%			
		10 days	5	64.00	89%	20.00	44.00	55	0	0	0	0	0	55	100.00%			
		10 days	5	287.90	96%	40.00	247.90	48	4	0	0	0	0	52	92.31%			
	discarded	10 days	5	144.00	94%	20.00	124.00	15	9	11	0	0	0	35	42.86%			
		10 days	5	54.00	85%	15.00	39.00	69	27	0	0	0	0	96	71.88%			
		10 days	5	52.80	85%	15.00	37.80	32	24	4	1	1	0	62	51.61%			
		10 days	5	76.80	95%	20.00	56.80	34	7	3	5	0	0	49	69.39%			
		10 days	5	31.20	98%	20.00	11.20	40	19	2	0	0	0	61	65.57%			
		10 days	5	377.50	95%	20.00	357.50	31	24	1	0	0	0	56	55.36%			
Leptotene	Batch1	16 days	8	165.00	87%	20.00	145.00	8	68	3	0	0	0	79	86.08%	145.00	87.00%	86.08%
	Batch2	16 days	6	212.00	85%	20.00	192.00	2	72	11	0	0	0	85	84.71%	192.00	85.00%	84.71%
	discarded	16 days	6	241.60	92%	20.00	221.60	14	30	8	0	0	0	52	26.92%			
		16 days	2	90.40	86%	20.00	70.40	17	19	9	0	0	0	45	37.78%			
		16 days	5	357.00	84%	15.00	342.00	13	17	2	0	0	0	32	40.63%			
Zygotene	Batch 1	2 weeks	8	160.00	92%	15.00	145.00	1	5	51	1	0	0	58	87.93%	804.40	87.30%	87.99%
		2 weeks	5	368.00	88%	15.00	353.00	9	10	131	1	0	0	151	86.75%			
		2 weeks	5	242.00	84%	20.00	222.00	11	3	140	1	0	0	155	90.32%			
		2 weeks	3	104.40	85%	20.00	84.40	5	3	54	0	0	0	62	87.10%			
	Batch 2	2 weeks	5	321.20	89%	20.00	301.20	4	5	42	0	0	0	51	82.35%	1137.40	89.18%	82.85%
		2 weeks	3	154.00	98%	15.00	139.00	17	4	97	3	0	1	122	79.51%			
		2 weeks	5	542.40	87%	20.00	522.40	12	4	93	2	0	0	111	83.78%			
		2 weeks	2	194.80	89%	20.00	174.80	11	0	66	2	0	0	79	83.54%			
	discarded	2 weeks	3	156.00	97%	15.00	141.00	6	8	32	1	0	0	47	68.09%			
		2 weeks	2	96.00	83%	15.00	81.00	0	3	8	0	0	0	11	72.73%			
		2 weeks	4	108.00	44%	20.00	88.00	12	8	31	0	0	0	51	60.78%			
		2 weeks	2	216.00	93%	20.00	196.00	16	6	33	0	0	0	55	60.00%			
		2 weeks	3	290.40	85%	15.00	275.40	24	2	96	2	2	0	126	76.19%			
		2 weeks	5	336.00	79%	15.00	321.00	31	4	91	0	1	0	127	71.65%			
		2 weeks	2	168.60	98%	20.00	148.60	15	7	22	0	0	0	44	50.00%			
Pachytene	Batch 1	5 weeks	2	34.70	95%	20.00	14.70	0	0	0	34	0	2	36	94.44%	400.68	87.19%	90.20%
		5 weeks	1	80.72	82%	20.00	60.72	0	0	0	20	0	1	21	95.24%			
		3 weeks	2	88.24	89%	20.00	68.24	0	0	9	58	0	1	68	85.29%			
		5 weeks	1	48.79	97%	20.00	28.79	0	0	0	37	2	0	39	94.87%			
		5 weeks	2	54.00	88%	20.00	34.00	0	0	1	35	1	0	37	94.59%			
		3 weeks	2	214.24	86%	20.00	194.24	0	0	2	31	2	0	35	88.57%			
	Batch 2	5 weeks	2	227.92	88%	20.00	207.92	0	0	0	10	0	0	10	100.00%	579.21	91.51%	92.97%
		5 weeks	1	117.76	92%	20.00	97.76	0	0	2	46	5	0	53	86.79%			
		5 weeks	2	293.53	94%	20.00	273.53	0	0	0	53	6	0	59	89.83%			
	discarded	5 weeks	2	132.94	73%	20.00	112.94	0	0	1	23	21	0	45	51.11%			
		5 weeks	1	51.48	69%	20.00	31.48	0	0	0	77	4	4	85	90.59%			
		5 weeks	1	74.88	89%	20.00	54.88	0	0	20	69	1	0	90	76.67%			
		3 weeks	4	127.98	87%	20.00	107.98	0	0	1	16	27	0	44	36.36%			
Diplotene	Batch 1	5 weeks	4	182.88	88%	20.00	162.88	0	0	0	0	27	0	27	100.00%	1052.75	88.63%	94.63%
		4 weeks	1	86.40	93%	20.00	66.40	0	0	0	1	41	1	43	95.35%			
		5 weeks	2	121.98	84%	20.00	101.98	0	0	0	0	50	0	50	100.00%			
		3 weeks	2	168.43	95%	20.00	148.43	0	0	0	1	40	0	41	97.56%			
		5 weeks	2	174.72	89%	20.00	154.72	0	0	1	13	59	0	73	80.82%			
		5 weeks	1	58.84	86%	20.00	38.84	0	0	3	1	58	0	62	93.55%			
		5 weeks	2	399.50	87%	20.00	379.50	0	0	1	0	41	1	43	95.35%			
	Batch 2	5 weeks	2	179.82	94%	20.00	159.82	0	0	0	0	38	0	38	100.00%	982.36	90.85%	96.13%
		5 weeks	2	203.36	84%	20.00	183.36	0	0	0	0	72	1	73	98.63%			
		5 weeks	2	105.30	82%	20.00	85.30	0	0	4	1	24	0	29	82.76%			
		5 weeks	2	213.38	97%	20.00	193.38	0	0	0	0	17	0	17	100.00%			
		5 weeks	2	243.46	94%	20.00	223.46	0	0	1	0	16	0	17	94.12%			
		5 weeks	1	157.04	88%	20.00	137.04	0	0	0	0	17	1	18	94.44%			
	discarded	5 weeks	1	42.44	36%	20.00	22.44	0	0	0	14	46	0	60	76.67%			
		5 weeks	1	98.28	94%	20.00	78.28	0	0	1	17	29	0	47	61.70%			
		5 weeks	1	91.46	91%	20.00	71.46	0	0	0	5	19	1	25	76.00%			
		5 weeks	2	109.67	85%	20.00	89.67	0	0	4	3	30	3	40	75.00%			
		5 weeks	1	94.28	82%	20.00	74.28	0	0	0	7	37	3	47	78.72%			

Table S2. Viability and Purity of isolated Spermatocytes for RNA-seq

Stage	Batch	Age of animals	Number of animals	Amount of cells(k)	Viability	Amount for spreading(k)	Microscopy counts of each stage: Preleptotene	Leptotene	Zygotene	Pachytene	Total	Purity	Amount for RNA-seq(k)
Preleptotene	Batch 1	10 days	3	80.00	96%	15.00	61	12	0	0	73	83.56%	65.00
	Batch 2	10 days	3	63.00	96%	15.00	47	0	0	0	47	100.00%	48.00
	discarded	10 days	3	73.20	92%	15.00	17	17	0	0	34	50.00%	
		10 days	5	162.00	94%	15.00	15	7	1	0	23	65.22%	
		10 days	3	126.40	92%	15.00	21	16	1	0	38	55.26%	
		10 days	3	50.00	86%	15.00	25	24	19	0	68	36.76%	
Leptotene	Batch1	16 days	5	138.00	96%	15.00	9	83	1	0	93	89.25%	123.00
	Batch2	16 days	2	61.20	91%	15.00	7	59	4	0	70	84.29%	46.20
	discarded	16 days	3	103.00	89%	15.00	10	27	0	0	37	72.97%	
		16 days	3	88.00	93%	15.00	25	38	2	0	65	58.46%	
Zygotene	Batch 1	2 weeks	2	86.00	96%	15.00	0	11	55	0	66	83.33%	71.00
	Batch 2	2 weeks	2	70.00	92%	15.00	0	12	74	0	86	86.05%	55.00
	discarded	2 weeks	2	102.00	95%	15.00	12	15	33	0	60	55.00%	
		2 weeks	2	50.00	90%	15.00	3	13	41	4	61	67.21%	
		2 weeks	2	46.00	89%	15.00	5	21	55	1	82	67.07%	

Table S3. Mapping statistics and data quality of Hi-C datasets

	Batch	Total Reads	Side 1 Aligned	Side 2 Aligned	Both Sides Aligned	Same Fragment	Dangling Ends	Self Circles	Error Pairs	valid pairs	% valid Pairs	Unique Valid Pairs	% Unique Valid Pairs	cis Pairs	% cis Pairs
**Preleptotene**	replicate 1	788,485,368	711,665,213	699,991,317	623,171,162	327,320,472	326,710,564	145,435	464,473	295,850,690	47.48%	213,461,110	34.25%	126,517,652	59.27%
	replicate 2	360,622,967	321,899,608	314,649,639	275,926,280	132,245,547	132,089,776	70,342	85,429	143,680,733	52.07%	124,061,390	44.96%	73,356,662	59.13%
**Leptotene**	replicate 1	455,995,942	408,920,694	407,636,070	360,560,822	207,781,151	207,636,248	68,186	76,717	152,779,671	42.37%	107,586,832	29.84%	65,450,431	60.83%
	replicate 2	630,782,691	563,375,572	561,235,532	493,828,413	271,370,429	271,194,707	77,546	98,176	222,457,984	45.05%	155,860,688	31.56%	95,062,938	60.99%
**Zygotene**	replicate 1	574,192,399	499,891,429	490,386,109	416,085,139	158,955,790	158,695,656	107,958	152,176	257,129,349	61.80%	222,006,481	53.36%	138,027,557	62.17%
	replicate 2	632,842,431	567,355,482	560,634,023	495,147,074	279,674,342	279,292,726	102,620	278,996	215,472,732	43.52%	159,763,148	32.27%	99,673,119	62.39%
**Pachytene**	replicate 1	601,282,672	518,453,847	510,223,666	427,394,841	136,216,119	135,778,637	279,260	158,222	291,178,722	68.13%	247,724,032	57.96%	139,242,295	56.21%
	replicate 2	604,847,535	526,951,648	512,714,152	434,818,265	105,018,709	102,255,501	376,698	2,386,510	329,799,556	75.85%	232,856,832	53.55%	130,497,699	56.04%
**Diplotene**	replicate 1	751,512,395	641,783,432	634,035,557	524,306,594	104,074,794	103,601,981	385,397	87,416	420,231,800	80.15%	367,535,195	70.10%	224,273,218	61.02%
	replicate 2	821,312,422	715,807,057	706,485,201	600,979,836	221,852,822	220,889,752	387,912	575,158	379,127,014	63.08%	326,799,047	54.38%	201,323,716	61.60%
**Meiosis II**	replicate 1	296,891,752	260,730,380	258,221,124	222,059,752	95,612,505	95,110,310	396,008	106,187	126,447,247	56.94%	99,506,870	44.81%	45,054,465	45.28%
	replicate 2	210,901,404	183,552,618	181,606,241	154,257,455	56,709,897	56,404,115	269,696	36,086	97,547,558	63.24%	81,266,625	52.68%	37,983,339	46.74%
**Spermatogonia**	replicate 1	208,420,384	183,448,696	180,714,481	155,742,793	78,897,203	78,564,998	116,267	215,938	76,845,590	49.34%	60,128,746	38.61%	32,590,807	54.20%
	replicate 2	134,683,325	108,471,087	108,299,369	82,087,131	12,141,961	11,925,917	174,770	41,274	69,945,170	85.21%	57,924,530	70.56%	33,326,234	57.53%
**Sertoli**	replicate 1	370,735,089	328,360,365	322,087,377	279,712,653	139,723,726	139,421,473	164,902	137,351	139,988,927	50.05%	117,088,802	41.86%	67,365,900	57.53%
	replicate 2	251,646,268	216,120,662	214,863,572	179,337,966	67,596,760	67,392,445	136,409	67,906	111,741,206	62.31%	92,073,032	51.34%	54,956,137	59.69%
**Sun1 mutant zygotene**	replicate 1	243,407,551	218,281,766	217,355,690	192,229,905	119,122,746	118,971,534	52,916	98,296	73,107,159	38.03%	59,469,277	30.94%	39,013,816	65.60%
	replicate 2	244,901,922	219,138,994	217,555,910	191,792,982	116,455,480	116,319,594	45,365	90,521	75,337,502	39.28%	62,551,017	32.61%	41,671,604	66.62%

Table S4 Primers for Hi-C libraries Construction

**Name**	**Sequence**	**Type**
**TruSeq_Adapter-Index01**	/5Phos/GATCGGAAGAGCACACGTCTGAACTCCAGTCACATCACGATCTCGTATGCCGTCTTCTGCTTG	Barcoded adaptor
**TruSeq_Adapter-Index02**	/5Phos/GATCGGAAGAGCACACGTCTGAACTCCAGTCACCGATGTATCTCGTATGCCGTCTTCTGCTTG	Barcoded adaptor
**TruSeq_Adapter-Index03**	/5Phos/GATCGGAAGAGCACACGTCTGAACTCCAGTCACTTAGGCATCTCGTATGCCGTCTTCTGCTTG	Barcoded adaptor
**TruSeq_Adapter-Index04**	/5Phos/GATCGGAAGAGCACACGTCTGAACTCCAGTCACTGACCAATCTCGTATGCCGTCTTCTGCTTG	Barcoded adaptor
**TruSeq_Adapter-Index05**	/5Phos/GATCGGAAGAGCACACGTCTGAACTCCAGTCACACAGTGATCTCGTATGCCGTCTTCTGCTTG	Barcoded adaptor
**TruSeq_Adapter-Index06**	/5Phos/GATCGGAAGAGCACACGTCTGAACTCCAGTCACGCCAATATCTCGTATGCCGTCTTCTGCTTG	Barcoded adaptor
**TruSeq _Adapter-Universal**	AATGATACGGCGACCACCGAGATCTACACTCTTTCCCTACACGACGCTCTTCCGATC/phosphorothioate bond/T	Universal adaptor
**TruSeq-PE1.0**	CAAGCAGAAGACGGCATACGAGA/phosphorothioate bond/T	Amplification primer
**TruSeq-PE2.0**	AATGATACGGCGACCACCGAGATCTACACTCTTTCCCTACACG/phosphorothioate bond/A	Amplification primer
