# Supplementary material for: Stage-resolved Hi-C analyses reveal meiotic chromosome organizational features influencing homolog alignment
Source: Nat Commun. 2021 Oct 8;12:5827. doi: 10.1038/s41467-021-26033-0 (PMC8501046; doi:10.1038/s41467-021-26033-0)
Supplement: Supplementary file 3 — Reporting Summary [file 41467_2021_26033_MOESM3_ESM.pdf]

nature research

Corresponding author(s): Qian Bian, Ming Lei

Last updated by author(s): Aug 30, 2021

Reporting Summary

Nature Research wishes to improve the reproducibility of the work that we publish. This form provides structure for consistency and transparency in reporting. For further information on Nature Research policies, see our Editorial Policies and the Editorial Policy Checklist.

Statistics

For all statistical analyses, confirm that the following items are present in the figure legend, table legend, main text, or Methods section.

n/a	Confirmed	
☐	☒	The exact sample size (n) for each experimental group/condition, given as a discrete number and unit of measurement
☐	☒	A statement on whether measurements were taken from distinct samples or whether the same sample was measured repeatedly
☐	☒	The statistical test(s) used AND whether they are one- or two-sided<br>*Only common tests should be described solely by name; describe more complex techniques in the Methods section.*
☒	☐	A description of all covariates tested
☒	☐	A description of any assumptions or corrections, such as tests of normality and adjustment for multiple comparisons
☐	☒	A full description of the statistical parameters including central tendency (e.g. means) or other basic estimates (e.g. regression coefficient) AND variation (e.g. standard deviation) or associated estimates of uncertainty (e.g. confidence intervals)
☐	☒	For null hypothesis testing, the test statistic (e.g. F, t, r) with confidence intervals, effect sizes, degrees of freedom and P value noted<br>*Give P values as exact values whenever suitable.*
☒	☐	For Bayesian analysis, information on the choice of priors and Markov chain Monte Carlo settings
☒	☐	For hierarchical and complex designs, identification of the appropriate level for tests and full reporting of outcomes
☐	☒	Estimates of effect sizes (e.g. Cohen's d, Pearson's r), indicating how they were calculated

Our web collection on statistics for biologists contains articles on many of the points above.

Software and code

Policy information about availability of computer code

Data collection	Sequencing data were collected on an Illumina Novaseq6000 with NovaSeq 6000 v1.5 Reagent Kits. Confocal images were taken by Leica TCS SP8 STED 3X with Leica application suite X (v3.7.0) and Zeiss LSM 880 confocal microscope with Zeiss Zen software (2012 S4).
Data analysis	Hi-C:<br>Hi-C reads were mapped to the mm10 genome and filtered using the hiclib data processing pipeline (https://bitbucket.org/mirnylab/hiclib, the version information is unavailable). The mapped Hi-C fragments of replicates were combined and converted into cooler format using the cooler package (https://github.com/mirnylab/cooler, v0.8.5). The cis and trans expected contact probability at different genomic separation was calculated using the cooltools package (https://github.com/mirnylab/cooltools, v0.2.0). Pearson correlation coefficients between Hi-C replicates for each stage or between datasets of different stages were calculated using cool files binned at 500kb and the HiCRep package (https://github.com/TaoYang-dev/hicrep, v1.11.0). Pileup analysis of chromatin loops was performed on Hi-C datasets binned at 10kb resolution using the coolpuppy package (https://github.com/Phlya/coolpuppy, v0.9.5). The GSE122622_zygonema_interhomolog.hic file was then converted to cooler format using the hic2cool package (https://github.com/4dn-dcic/ hic2cool, v0.8.3).<br><br>RNA-seq:<br>All RNA-seq raw data were trimmed to remove low-quality reads and adapters with Trim Galore (v0.6.5). The clean reads were then mapped to the mouse reference genome (mm10, https://hgdownload.soe.ucsc.edu/goldenPath/archive/mm10/) using the STAR aligner (v2.7.3a) with default parameters and the annotated genes were counted by HTSeq (v0.12.4). The raw counts files were normalized using the DESeq2 (v1.22.2) package and heatmaps were generated using the ggplot2 (v3.3.0) and pheatmap (v1.0.12) packages in R. Coverage tracks at 10bp and 100kb bin sizes were generated using the bamCoverage (v3.3.1) utility from the deeptools (v3.3.1) package with the parameter "-normalizeUsing RPKM".

Cut&Tag:
Low-quality reads and adapters were trimmed by Trim Galore (v0.6.5). The clean reads were mapped to the mm10 genome with bowtie2 (v2.4.2) and duplicates were removed with Picard (http://broadinstitute.github.io/picard/, v2.18.29). Peaks were called using the MACS2 (v2.1.2) callpeak function with input data as control.

Microscopic Images:
The image were analyzed with Leica application suite X (v3.7.0) and with Zeiss Zen software (2012 S4), the FACS profiles were analyzed with BD FACSTM Software 1.2.0.142.

Code availability:
Custom scripts used in this study are publicly available at https://github.com/bianlab-hub/zuo_ncomms_2021/tree/Hi-C_data_analysis with DOI: https://doi.org/10.5281/zenodo.5282919.

For manuscripts utilizing custom algorithms or software that are central to the research but not yet described in published literature, software must be made available to editors and reviewers. We strongly encourage code deposition in a community repository (e.g. GitHub). See the Nature Research guidelines for submitting code & software for further information.

Data

Policy information about availability of data

All manuscripts must include a data availability statement. This statement should provide the following information, where applicable:

- Accession codes, unique identifiers, or web links for publicly available datasets
- A list of figures that have associated raw data
- A description of any restrictions on data availability

All raw data of high-throughput sequencing and processed files for wild-type cells used in this study have been deposited in the National Center for Biotechnology Information (NCBI) Gene Expression Omnibus (GEO) under the accession codes GSE155638 [https://www.ncbi.nlm.nih.gov/geo/query/acc.cgi?acc=GSE155638] and GSE155967 [https://www.ncbi.nlm.nih.gov/geo/query/acc.cgi?acc=GSE155967]. The Hi-C data for SUN1 W151R mutant zygotene spermatocytes have been deposited in GEO under the accession code GSE155142 [https://www.ncbi.nlm.nih.gov/geo/query/acc.cgi?acc=GSE155142]. The following publicly available datasets used in the manuscript were downloaded using GEO platform: Inter-homolog Hi-C data for zygotene stage spermatocytes (GSE122622, [https://www.ncbi.nlm.nih.gov/geo/query/acc.cgi?acc=GSE122622]); Pachytene/ diplotene stage CTCF and REC8 ChIP-Seq data (GSE132054, [https://www.ncbi.nlm.nih.gov/geo/query/acc.cgi?acc=GSE132054]). Reference genome data of mouse was mm10 (https://hgdownload.soe.ucsc.edu/goldenPath/archive/mm10/).

Field-specific reporting

Please select the one below that is the best fit for your research. If you are not sure, read the appropriate sections before making your selection.

- [x] Life sciences
- [ ] Behavioural & social sciences
- [ ] Ecological, evolutionary & environmental sciences

For a reference copy of the document with all sections, see nature.com/documents/nr-reporting-summary-flat.pdf

Life sciences study design

All studies must disclose on these points even when the disclosure is negative.

Sample size	The sample sizes were not determined by calculations. Two independent biological replicates of Hi-C and RNA-Seq experiments were performed in line with the ENCODE consortium experimental standards.
Data exclusions	No data were excluded from analysis.
Replication	The Hi-C and RNA-Seq experiments were performed on independent biological duplicates. The reproducibility between replicates was evaluated by performing several analyses that include calculating the Pearson correlation coefficients between the contact probability matrices and insulation profiles for Hi-C datasets and between the normalized gene count tables for RNA-Seq datasets. Overall, the biological replicates of the same cell type exhibit a higher degree of correlation with each other than with datasets of different cell types, suggesting that all attempts at replication were successful. The microscopic imaging experiments for assessing the arrangement of chromosome ends in Supplementary Figures 18 and 20 were performed on cells isolated from three independent FACS sorting experiments. All attempts at replication were successful for those experiments.
Randomization	For all the NGS and imaging experiments, the spermatocytes at different meiotic stages (e.g. leptotene, zygotene, pachytene, etc.) were isolated from multiple randomly selected mice of appropriate ages (Supplementary Table 1). The spermatocytes of the same meiotic stages were pooled together for subsequent Hi-C or RNA-Seq library preparation. Therefore, the allocation of animals for isolating different meiotic cell types was random.
Blinding	In this study, we performed Hi-C analysis on multiple wild-type (Sertoli, spermatogonia, preleptotene, leptotene, zygotene, pachytene, diplotene, and Meiosis II) cell types as well as the SUN1-Mutant zygotene spermatocytes. To ensure the purity of each cell type before the Hi-C library preparation, the isolated cells need to be assessed by microscopy based on the distinct chromosome morphology for different meiotic stages (Supplementary Figure 2). Therefore, blinding to different cell types was not applicable at the sample collection stage. After the Hi-C data analysis, the differences in Hi-C heatmaps for different wild-type and mutant meiotic cell types were so evident that blinding is not necessary.

Reporting for specific materials, systems and methods

We require information from authors about some types of materials, experimental systems and methods used in many studies. Here, indicate whether each material, system or method listed is relevant to your study. If you are not sure if a list item applies to your research, read the appropriate section before selecting a response.

Materials & experimental systems

n/a	Involved in the study
☐	☒ Antibodies
☒	☐ Eukaryotic cell lines
☒	☐ Palaeontology and archaeology
☐	☒ Animals and other organisms
☒	☐ Human research participants
☒	☐ Clinical data
☒	☐ Dual use research of concern

Methods

n/a	Involved in the study
☒	☐ ChIP-seq
☐	☒ Flow cytometry
☒	☐ MRI-based neuroimaging

Antibodies

Antibodies used

anti-SYCP3 (Abcam, ab15093)
anti-phospho-Histone γH2AX (Novus, NB100-384)
anti-FSHR (Abcam, ab113421)
anti-DMRT1 (Santa Cruz, sc-377167)
anti-CTCF antibody (Abcam, ab128873, Rabbit monoclonal [EPR7314(B)] to CTCF)
Rabbit IgG(Beyotime, A7016)
Goat anti-Rabbit Secondary Antibody, DyLight 550 (Thermo, 84541, AB_10942173)
Goat anti-Mouse Secondary Antibody, DyLight 488 (Thermo, 35502, AB_844397)

Validation

All the antibodies used in this study were validated by the manufacturers and have been used in previously published papers according to the manufacturer's website.

Anti-SYCP3 (Abcam, ab15093) has been validated for use in immunohistochemistry and Immunofluorescence, as stated on the Abcam product page, and has been referenced in 144 papers (https://www.abcam.com/SCP3-antibody-ab15093.html).

Anti-phospho-Histone γH2AX (Novus, NB100-384) has been validated for use in western blot, chromatin immunoprecipitation, immunohistochemistry and Immunofluorescence, as stated on the novusbio product page, and has been referenced in 111 papers (https://www.novusbio.com/products/gamma-h2ax-antibody_nb100-384#reviews-publications).

Anti-FSHR (Abcam, ab113421) has been validated for use in immunohistochemistry and Immunofluorescence, as stated on the Abcam product page, and has been referenced in 3 papers (https://www.abcam.com/fsh-r-antibody-ab113421.html).

Anti-DMRT1 (Santa Cruz, sc-377167) has been validated for use in western blot, immunoprecipitation, ELISA, immunohistochemistry and Immunofluorescence, as stated on the Santa Cruz product page, and has been referenced in 8 papers (https://www.scbt.com/p/dmrt1-antibody-a-9).

Anti-CTCF antibody (Abcam, ab128873, Rabbit monoclonal [EPR7314(B)] to CTCF) has been validated for use in western blot, chromatin immunoprecipitation, CUT&Tag-seq, immunohistochemistry and Immunofluorescence, as stated on the Abcam product page, and has been referenced in 11 papers (https://www.abcam.com/ctcf-antibody-epr7314b-chip-grade-ab128873.html).

Rabbit IgG (Beyotime, A7016) has been validated for use in western blot, chromatin immunoprecipitation, Co-immunoprecipitation (Co-IP), immunohistochemistry and Immunofluorescence, as stated on the Beyotime product page, and has been referenced in 52 papers (https://www.beyotime.com/product/A7016.htm).

Goat anti-Rabbit Secondary Antibody, DyLight 550 (Thermo, 84541, AB_10942173) has been validated for use in western blot, immunoprecipitation, FACS, immunohistochemistry and Immunofluorescence, as stated on the ThermoFisher product page, and has been referenced in 8 papers (https://www.thermofisher.com/antibody/product/Goat-anti-Rabbit-IgG-H-L-Secondary-Antibody-Polyclonal/84541).

Goat anti-Mouse Secondary Antibody, DyLight 488 (Thermo, 35502, AB_844397) has been validated for use in western blot, immunoprecipitation, FACS, immunohistochemistry and Immunofluorescence, as stated on the ThermoFisher product page, and has been referenced in 22 papers (https://www.thermofisher.com/antibody/product/Goat-anti-Mouse-IgG-H-L-Secondary-Antibody-Polyclonal/35502).

Animals and other organisms

Policy information about studies involving animals; ARRIVE guidelines recommended for reporting animal research

Laboratory animals	C57BL/6J (B6, Shanghai lab, Animal Research Center) male mice were housed under controlled environmental conditions with free access to water and food, with constant ambient temperature (22±2°C) and humidity (55±10%), and an alternating 12h light/dark cycle. C57BL/6J SUN1 W151R knock-in mice were generated by oocyte injection of CRISPR-Cas9 as described previously and housed under the same conditions as the wild-type mice. Experimental protocols were approved by the regional ethical committee of the National Center for Protein Science Shanghai. Every effort was made to minimize and refine the experiments to avoid animal suffering.
Wild animals	This study did not involve wild animals.
Field-collected samples	This study did not involve field-collected samples.
Ethics oversight	Experimental protocols were approved by the regional ethical committee of the National Center for Protein Science Shanghai.

Note that full information on the approval of the study protocol must also be provided in the manuscript.

Flow Cytometry

Plots

Confirm that:

- [x] The axis labels state the marker and fluorochrome used (e.g. CD4-FITC).
- [x] The axis scales are clearly visible. Include numbers along axes only for bottom left plot of group (a 'group' is an analysis of identical markers).
- [x] All plots are contour plots with outliers or pseudocolor plots.
- [x] A numerical value for number of cells or percentage (with statistics) is provided.

Methodology

Sample preparation	Wild-type or mutant mice of different ages were used for isolation of spermatocytes at different stages: 7-11 days old mice were used for isolating Sertoli, spermatogonia, and preleptotene cells, 2-week old mice for zygotene cells, 3- to 5-week-old mice for pachytene, diplotene, and Metaphase II, and 1-2 months old SUN1 W151R mutant mice for zygotene-like spermatocytes. Decapsulated testes were digested with Collagenase IV (1mg/ml)/ DNase I (5ug/ml) in DMEM at 37℃for about 15 min until the appearance of dispersed tubules. The tubules were then collected and digested with 0.025% trypsin at 37℃ for 15min. During digestion, the samples were gently pipetted every 5 min. Digestion was stopped by adding 4 volumes of DMEM with 10% FBS. Cells were collected by centrifugation, resuspended in complete DMEM, and filtered using a 70µm strainer. Cells were then stained with Hoechst 33342 (5µg /ml)/ DNase I (5µg /ml) at 37℃ for 1hr with gentle agitation every 10 min. Following staining, the cells were pelleted, resuspended in complete DMEM with propidium iodide (PI, 5µg/ml)/ Hoechst 33342/ DNase I, and filtered through a 40um strainer.
Instrument	BD Influx cell sorter and BD FACSCalibur Flow Cytometer
Software	BD FACSTM Software 1.2.0.142
Cell population abundance	~50-200 thousands of cells for the desired stage from each experiment can be sorted.
Gating strategy	Testicular cells were first gated by FSC-A vs SSC-A to eliminate any debris, then gated for singlets by FSC-H vs FSC-A or Trigger pulse width vs FSC-A. For live cell analysis, cells were further gated by PI staining to eliminate dead cells and spermatocytes were then distinguished by the fluorescent profiles of two emission channels of Hoechst (Hoechst Blue and Hoechst Red).

- [x] Tick this box to confirm that a figure exemplifying the gating strategy is provided in the Supplementary Information.
